# Supplementary material for: Soil water content drives the spatiotemporal the distribution and community assembly of soil ciliates in the Nianchu River Basin, Qinghai-Tibet Plateau, China
Source: PLoS One. 2024 Jul 10;19(7):e0299815. doi: 10.1371/journal.pone.0299815 (PMC11236148; doi:10.1371/journal.pone.0299815)
Supplement: S1 Fig — (DOCX) [file pone.0299815.s001.docx]

*Chilodonella bavariensis*


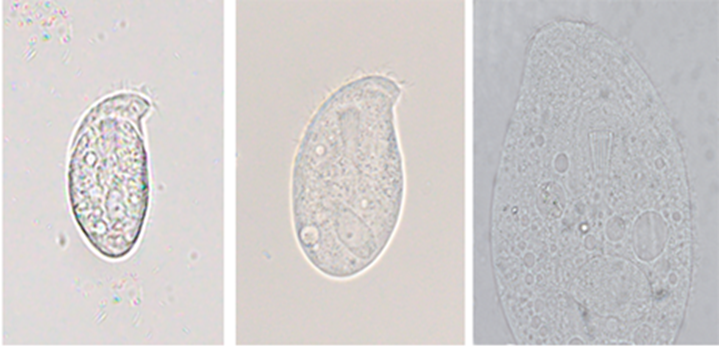


*Blepharisma steini*


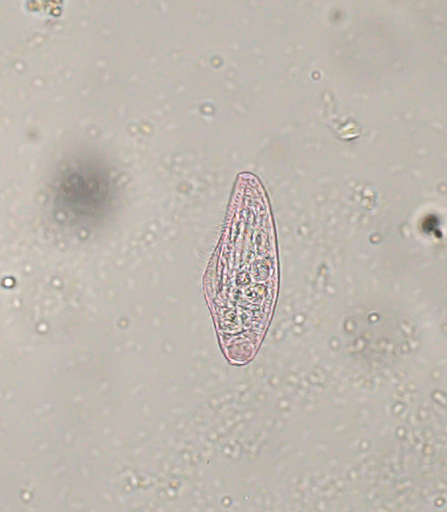


*Dileptus margaritifer*


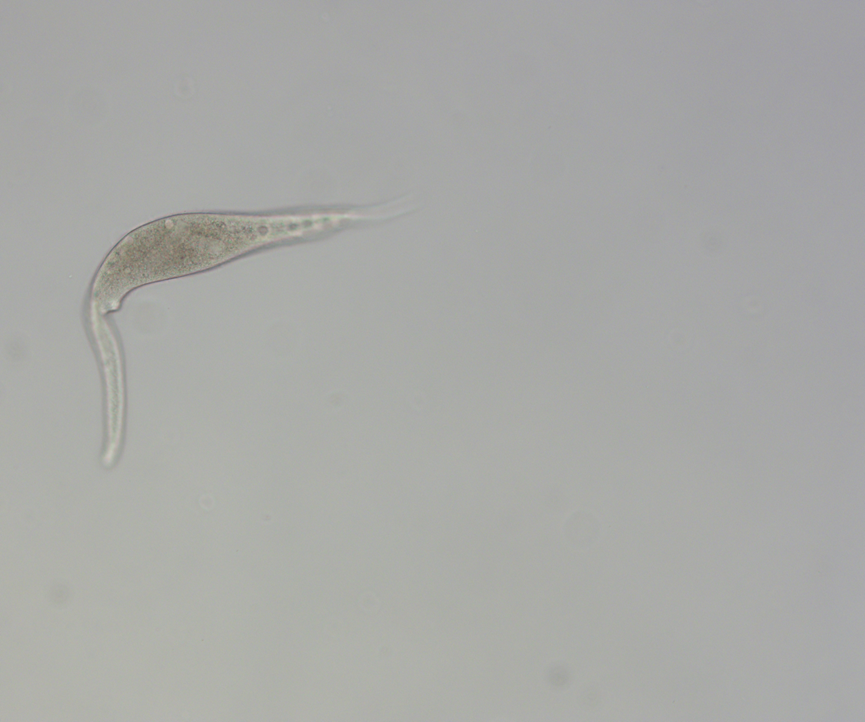


*Dileptus alpinus*


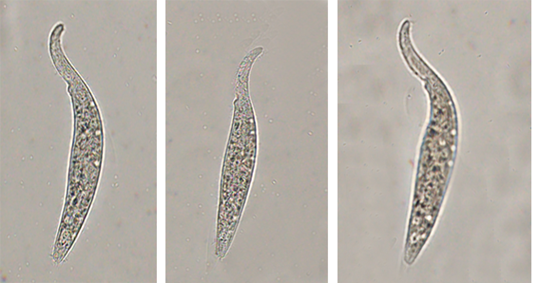


*Gonostomum a ffina*


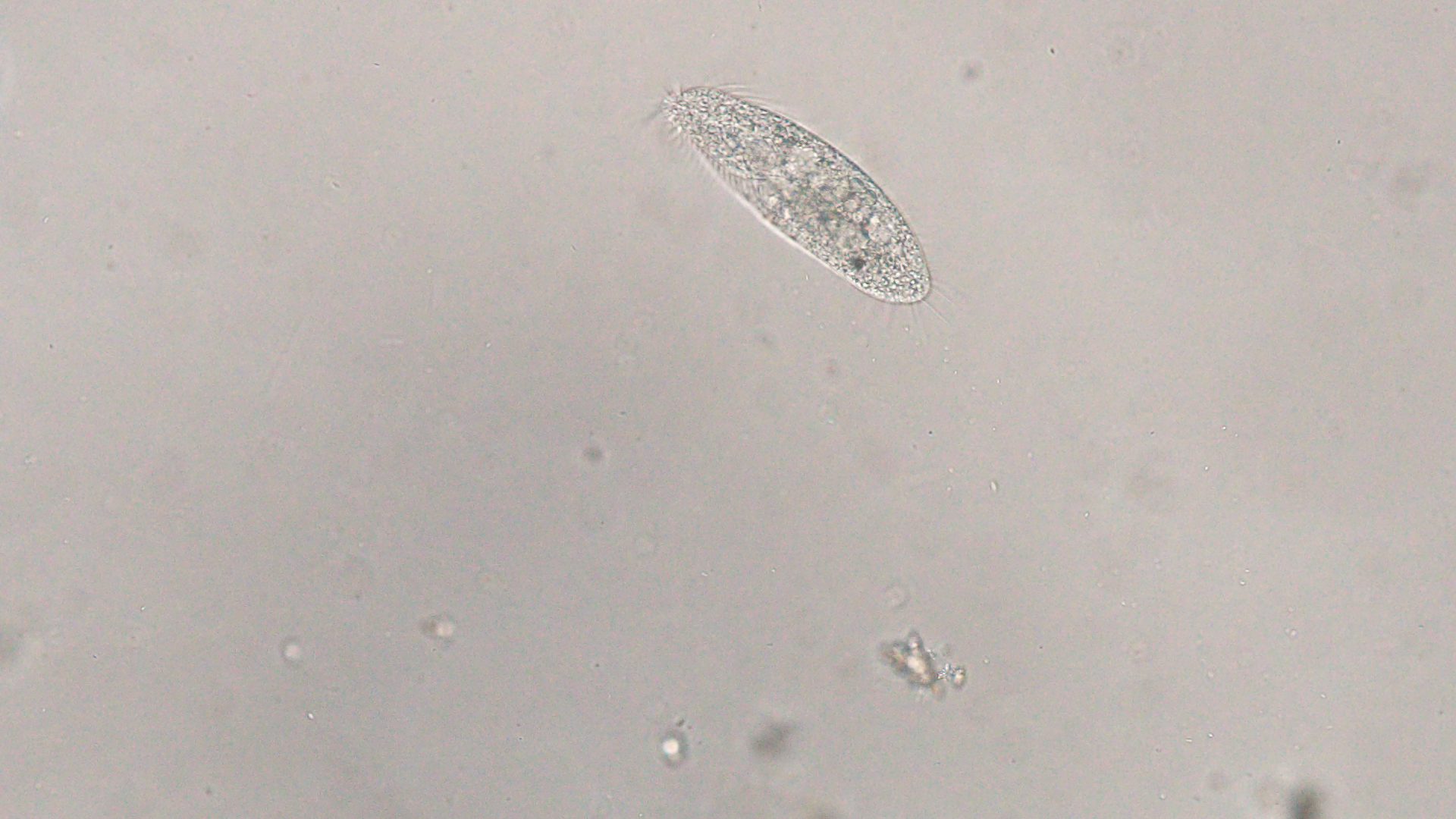

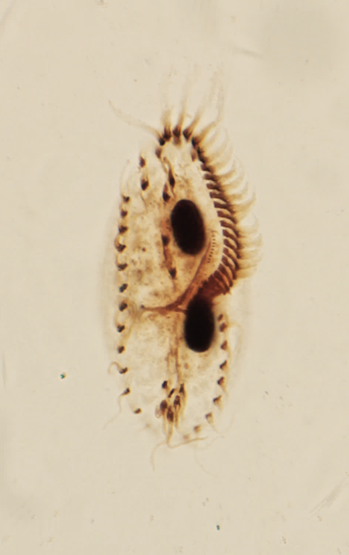


*Colpoda cucullus*


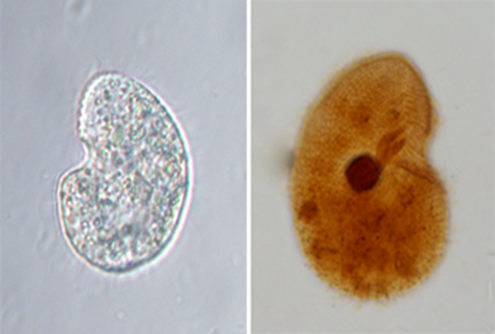


*Vorticella* sp.


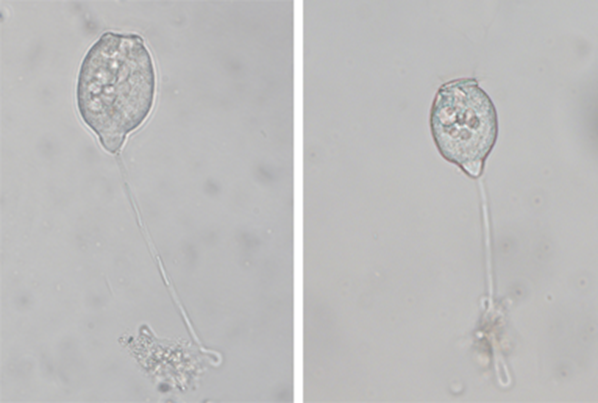


*Halteria grandinella*


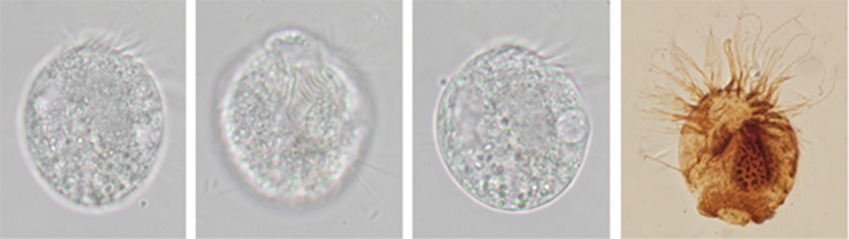


*Colpoda inflata*


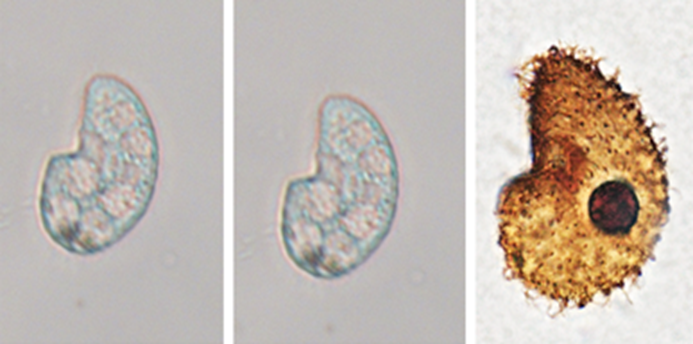


*Nassula aurea*


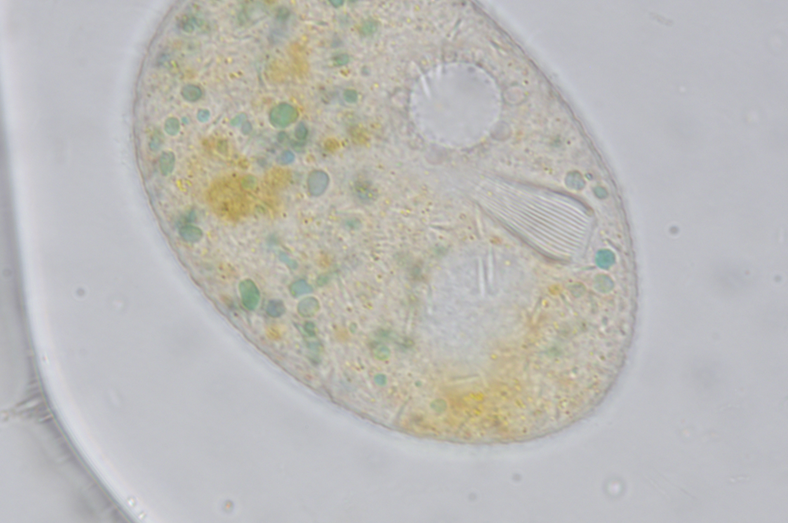


*Nassula ornata*


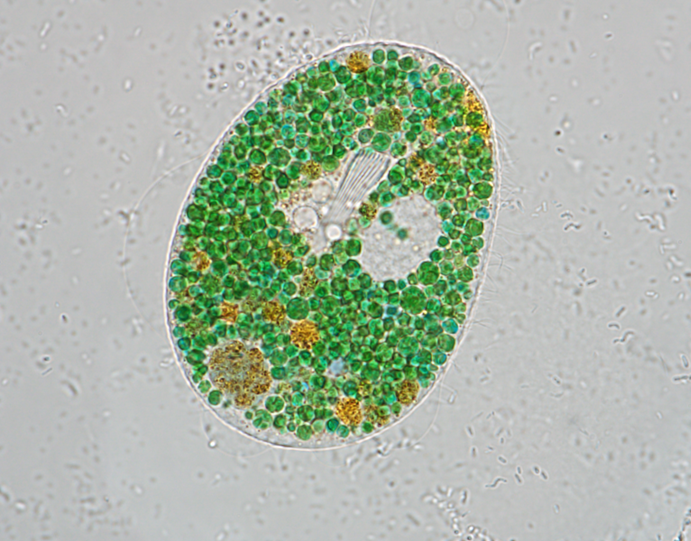


*Metopus rostratus*


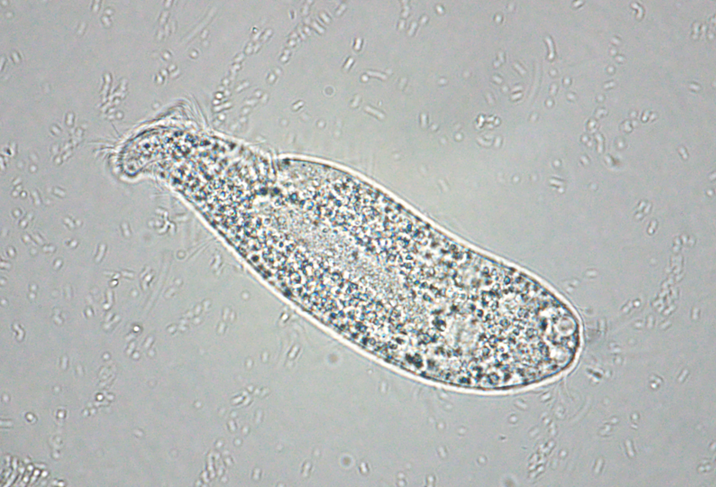


*Frontonia leucas*


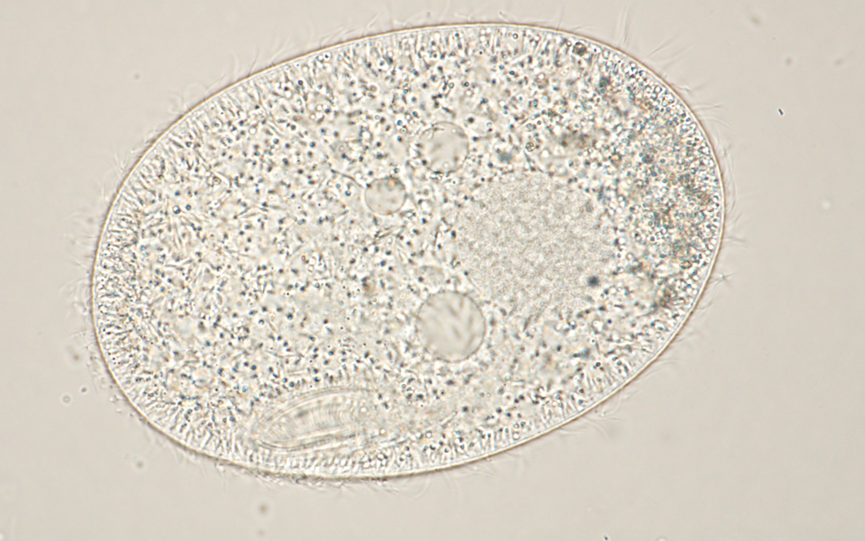


*Paracolpoda steini*


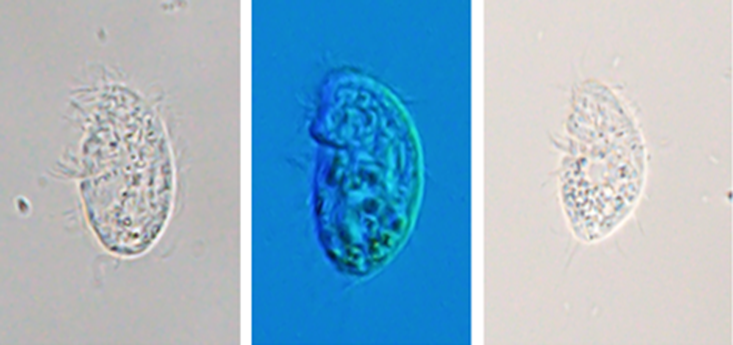


*Spathidium* sp*.*1


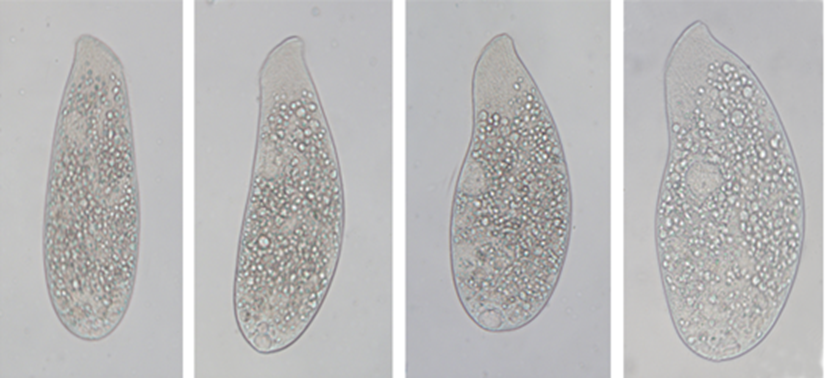


*Uroleptus caudatus*


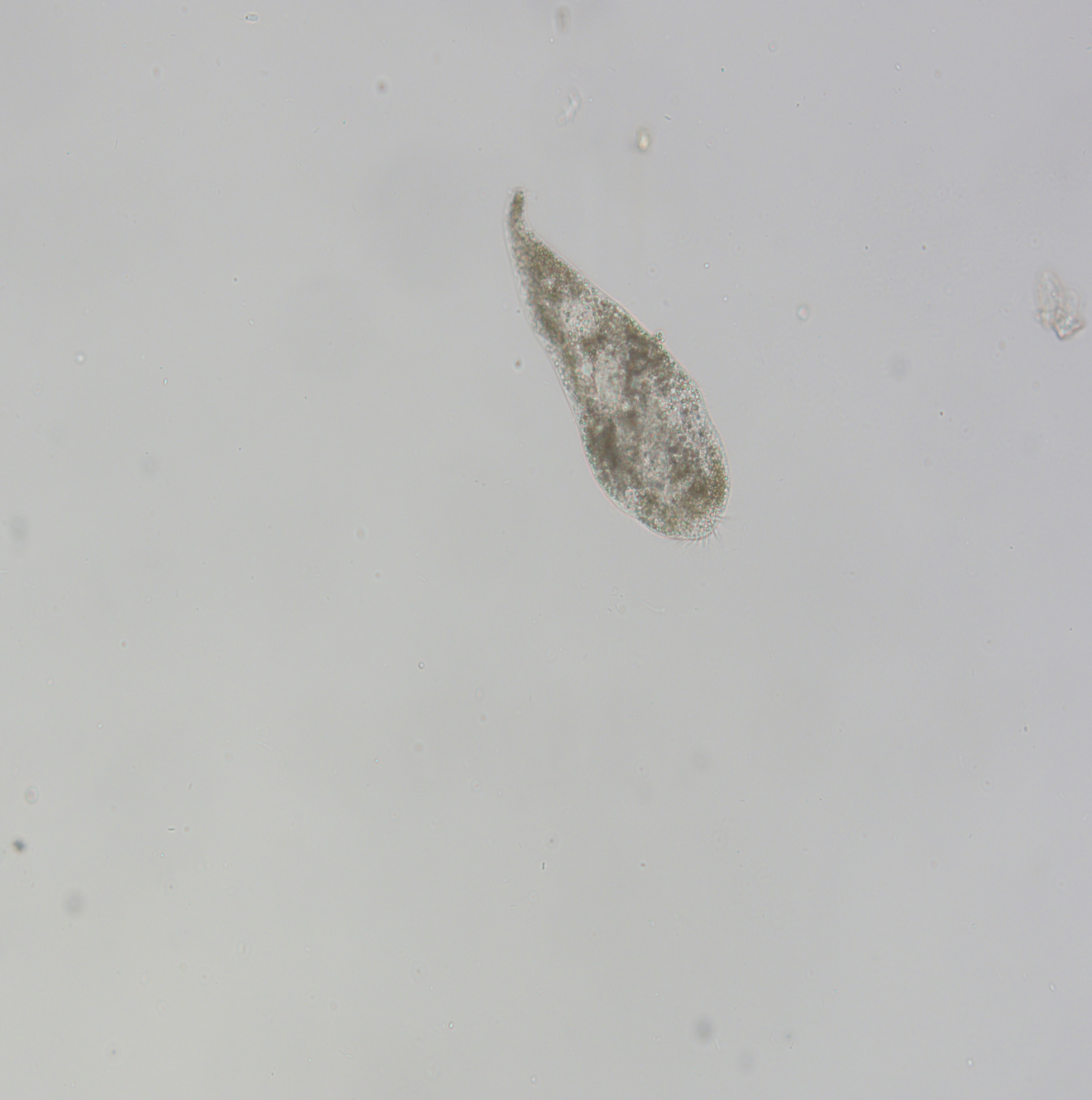

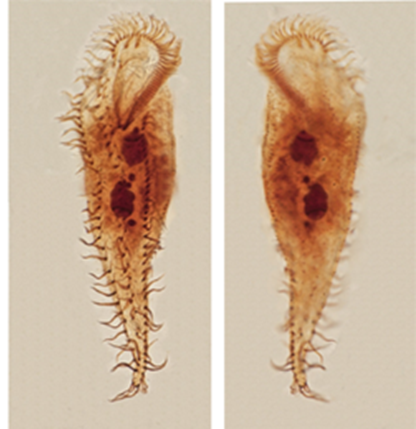


*Loxophyllum uninucleatum*


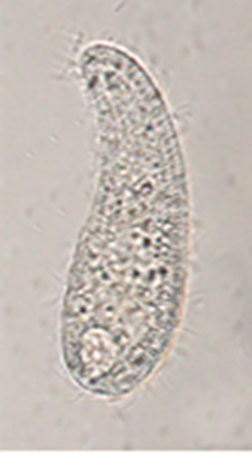


*Colpoda henneguyi*


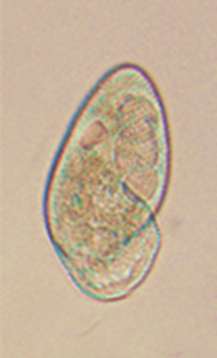


*Chilodontopsis vorax*


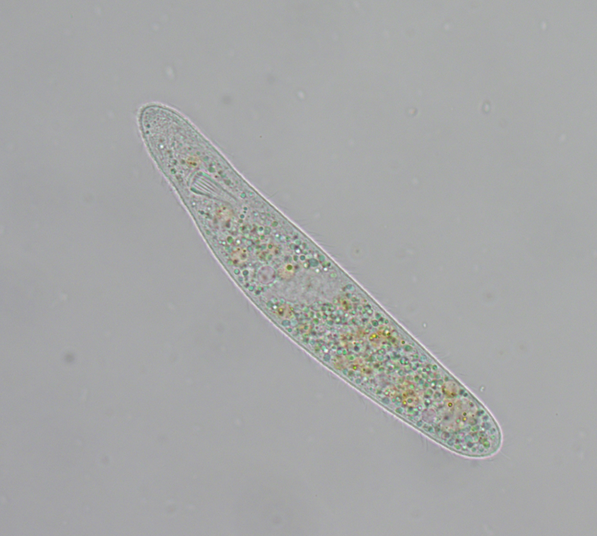


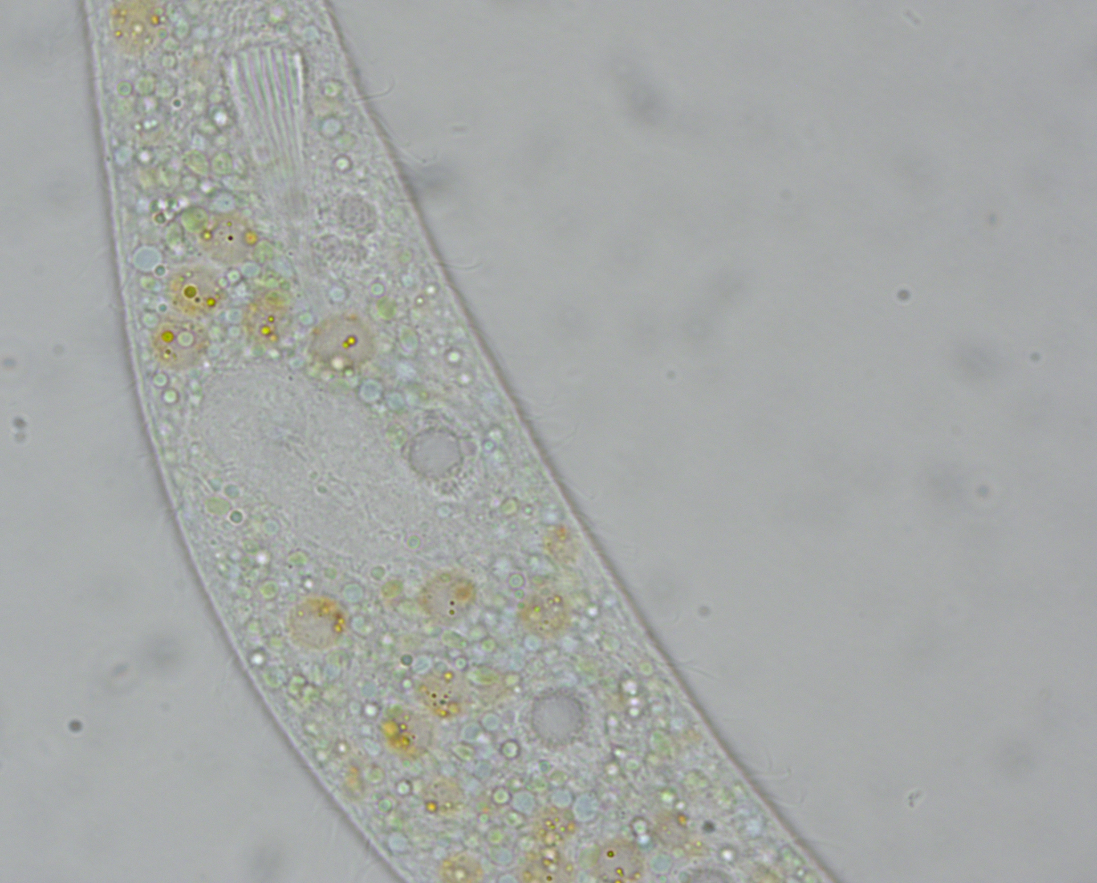


*Euplotes patella*


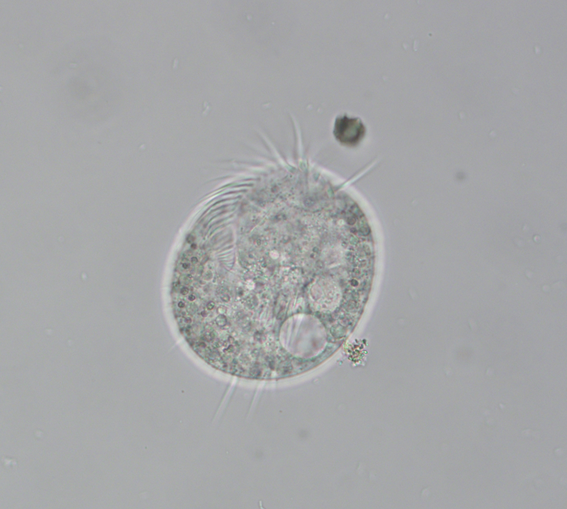


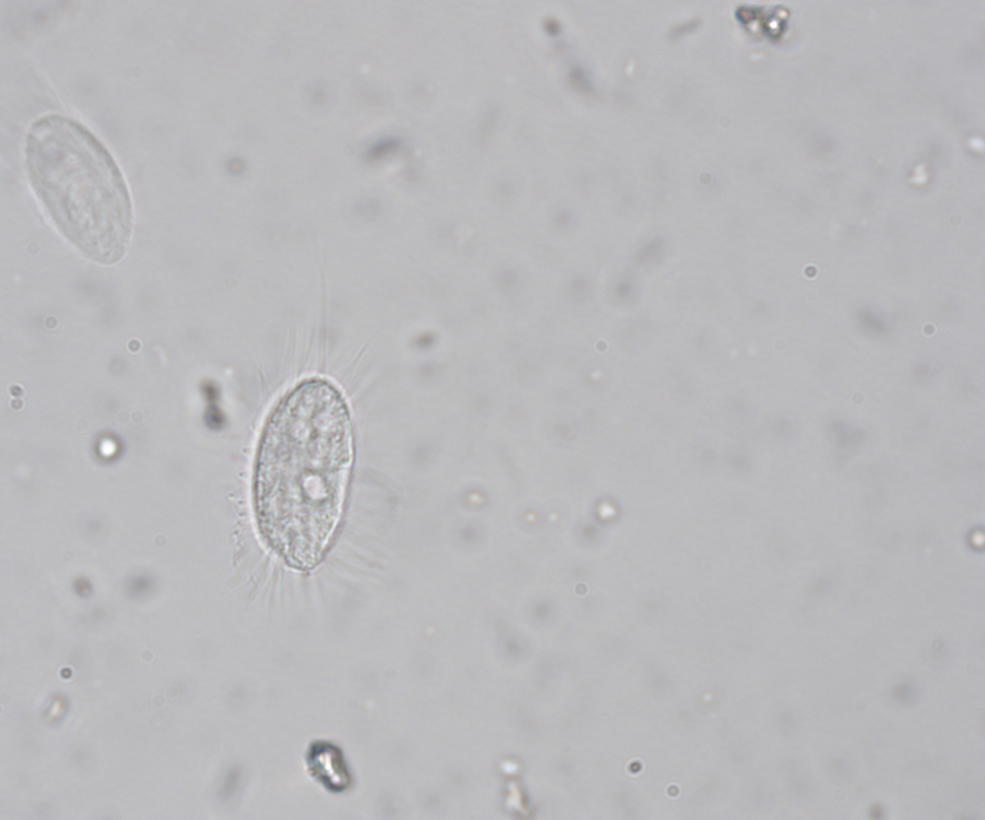

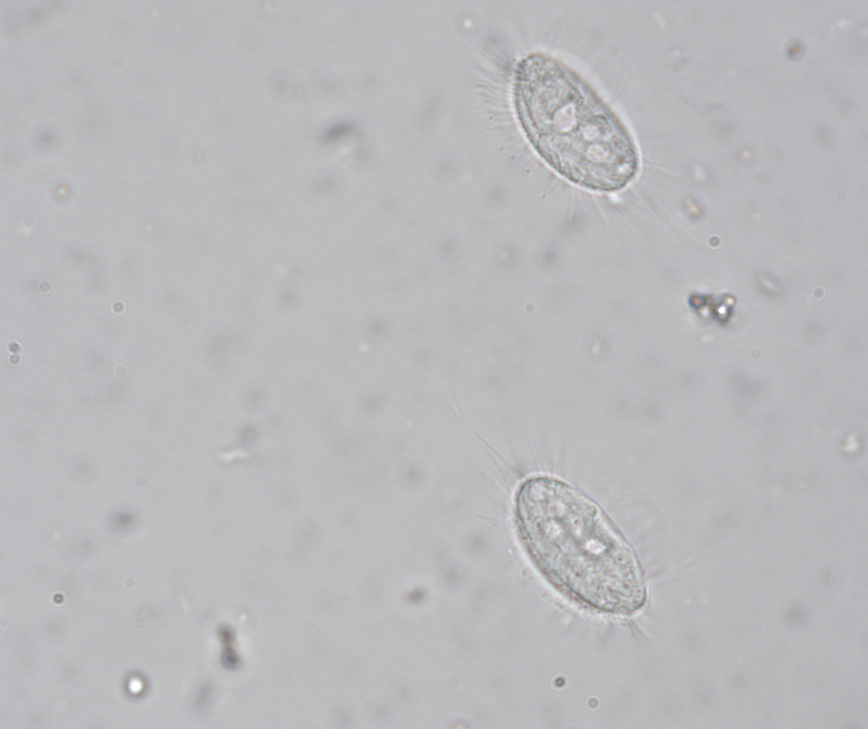


*Oxytricha fallax*


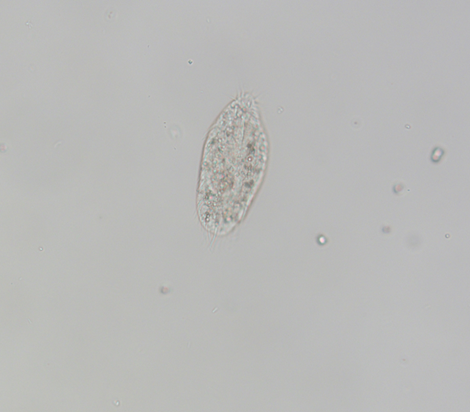


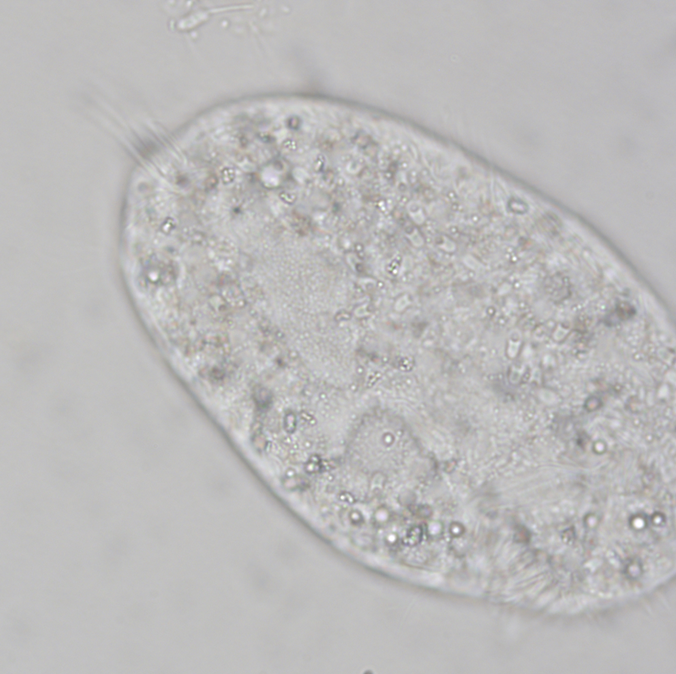


*Trachelophyllum chilense*


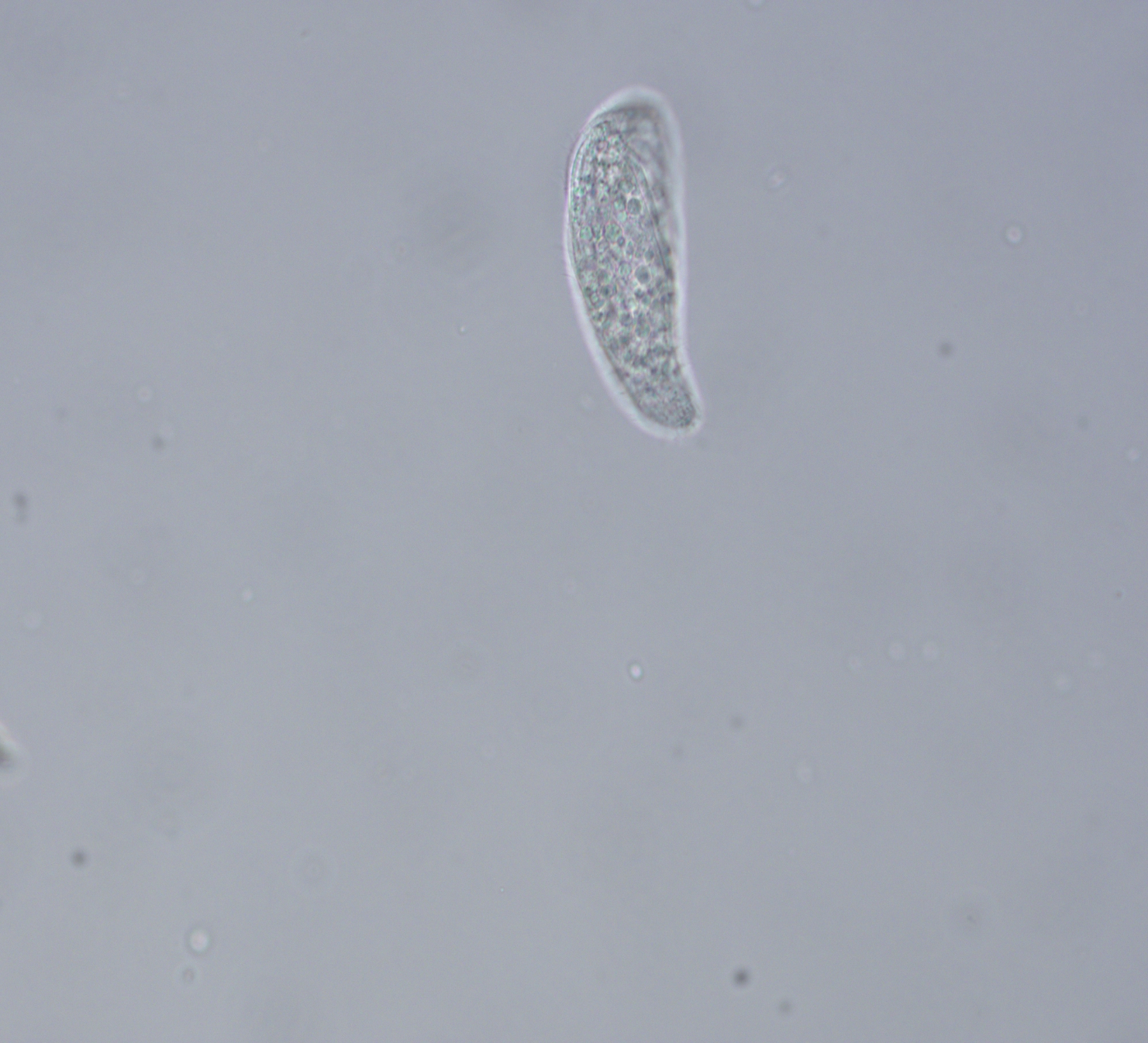

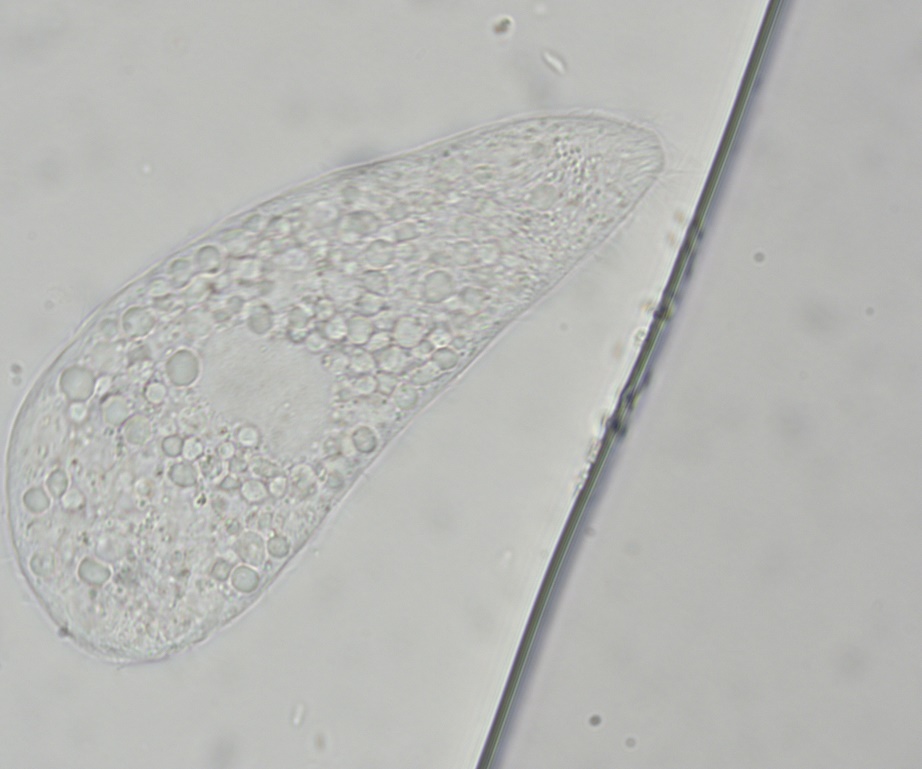


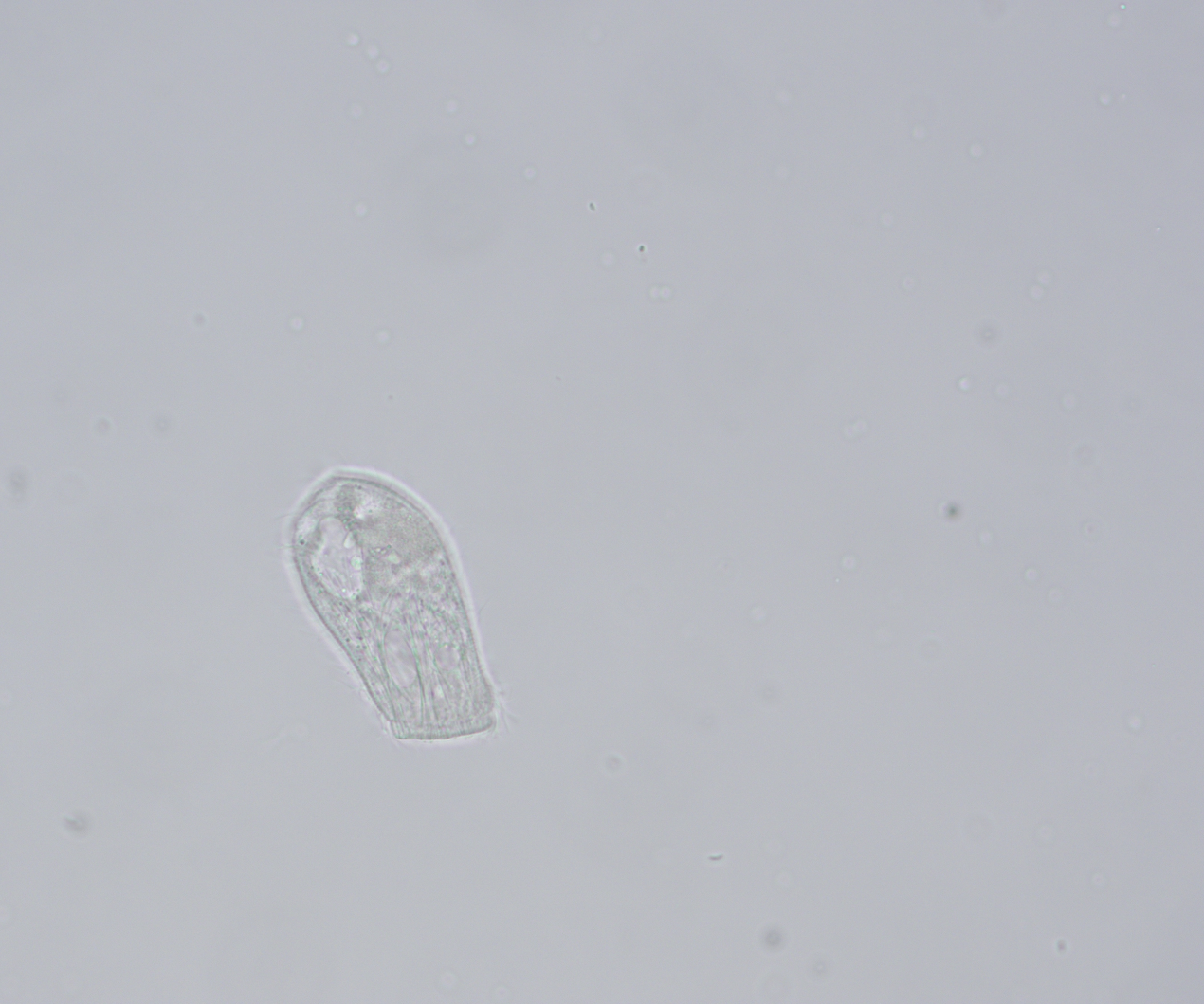

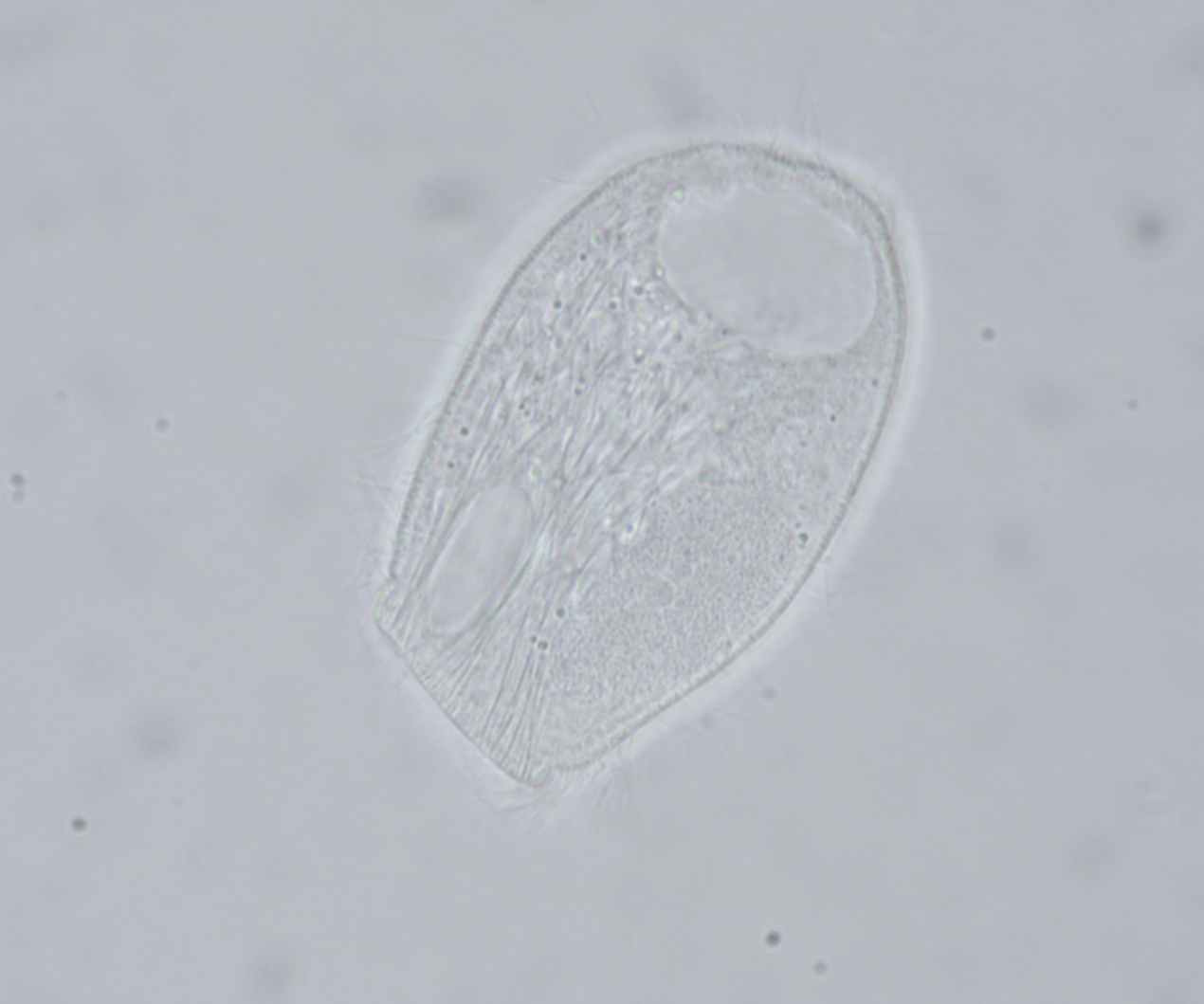


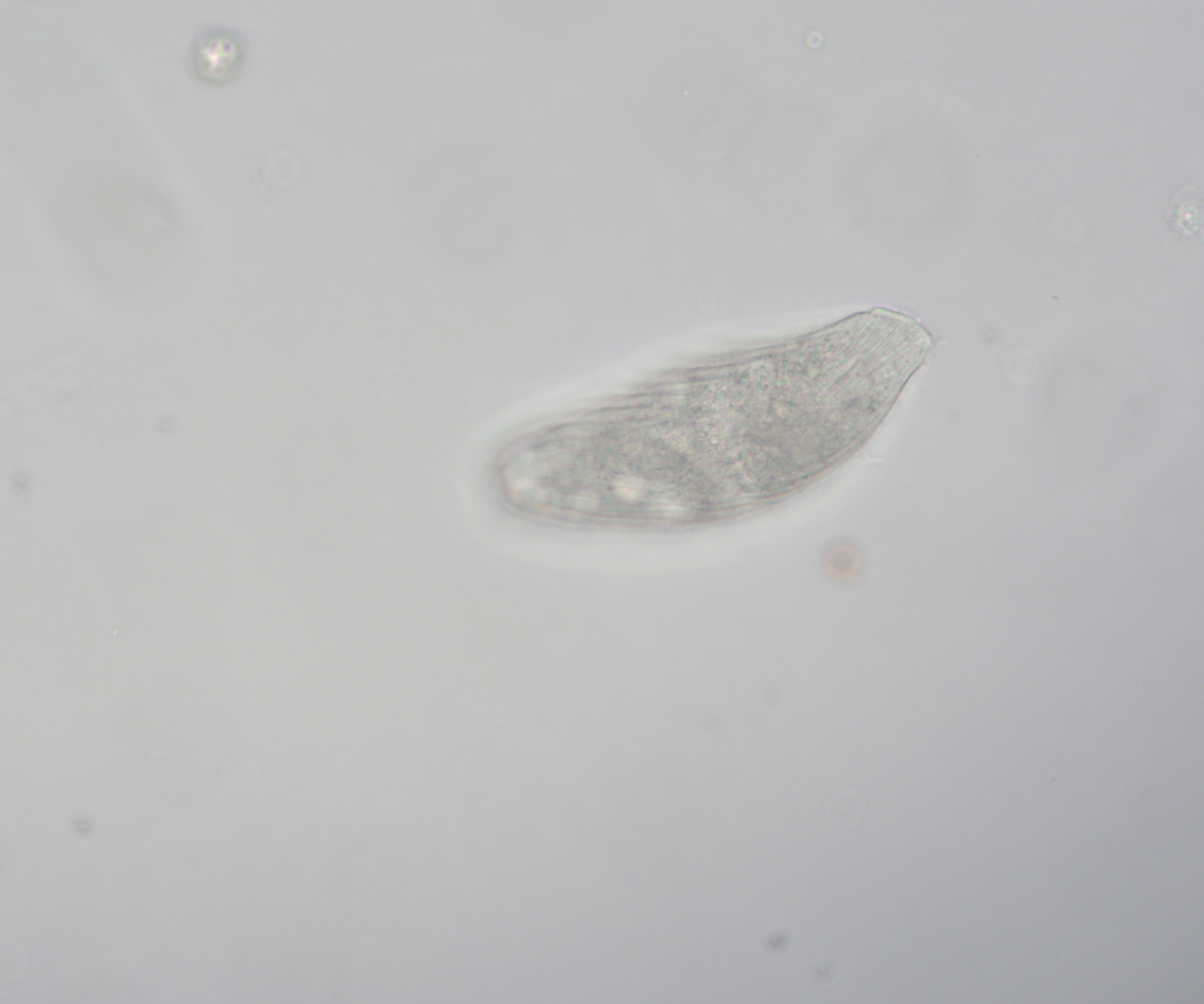


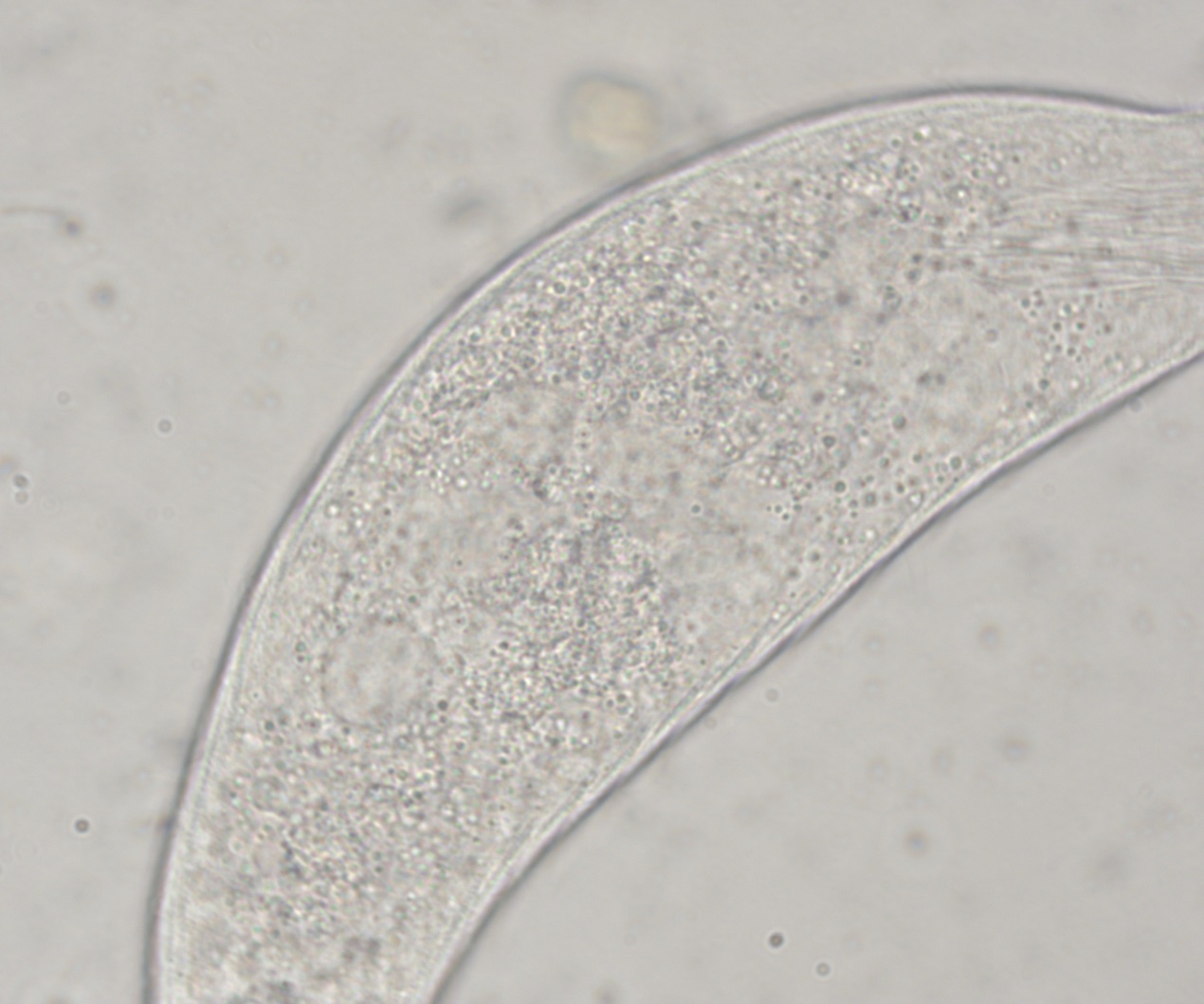


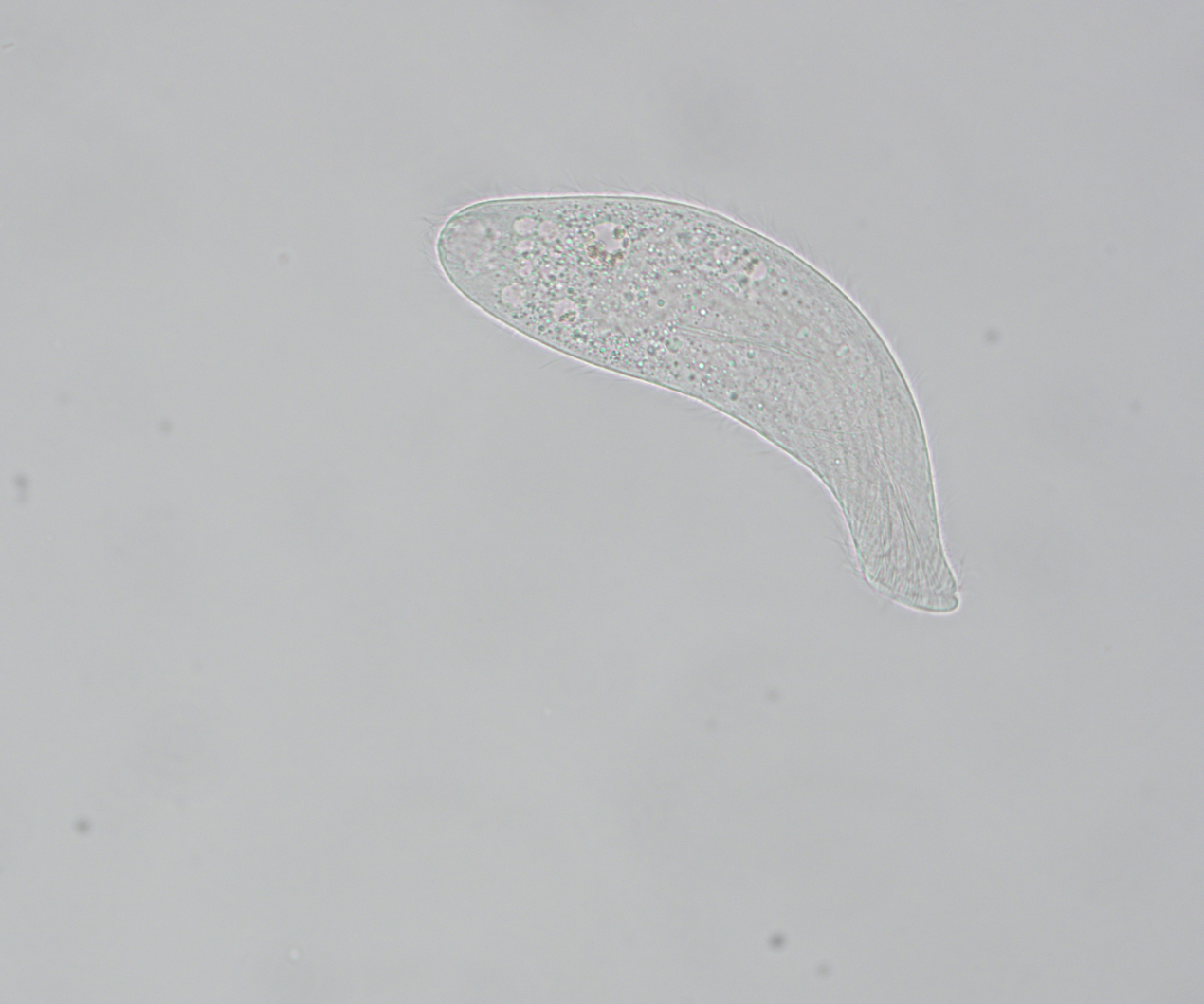

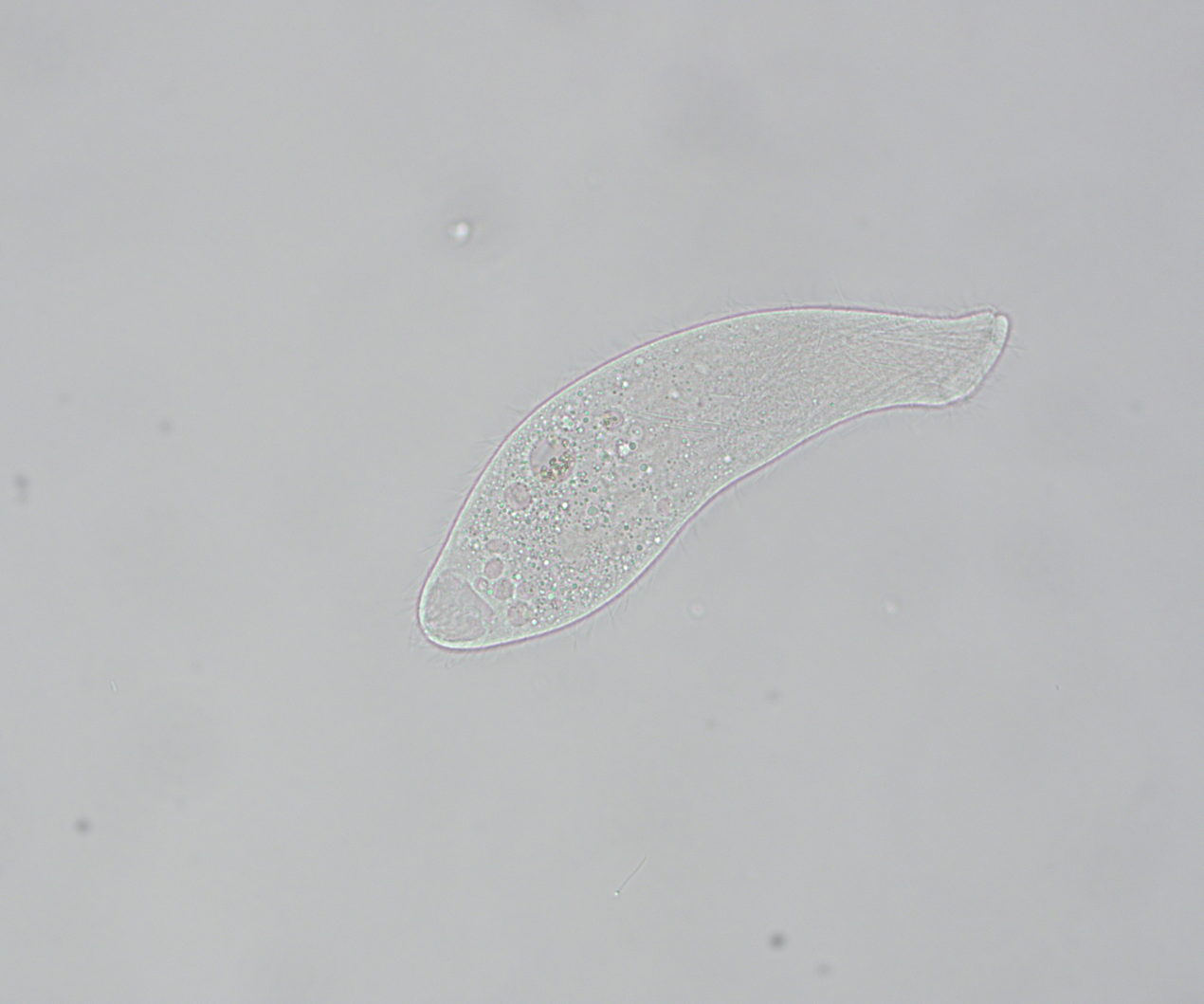


*Uroleptus dispar*


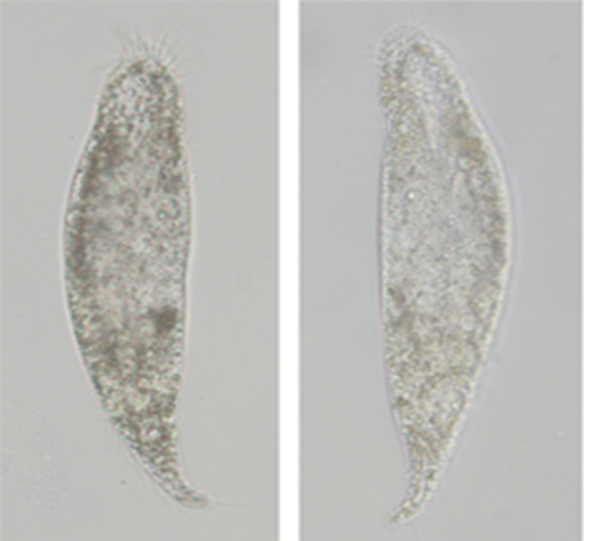


*Trachelius ovum*


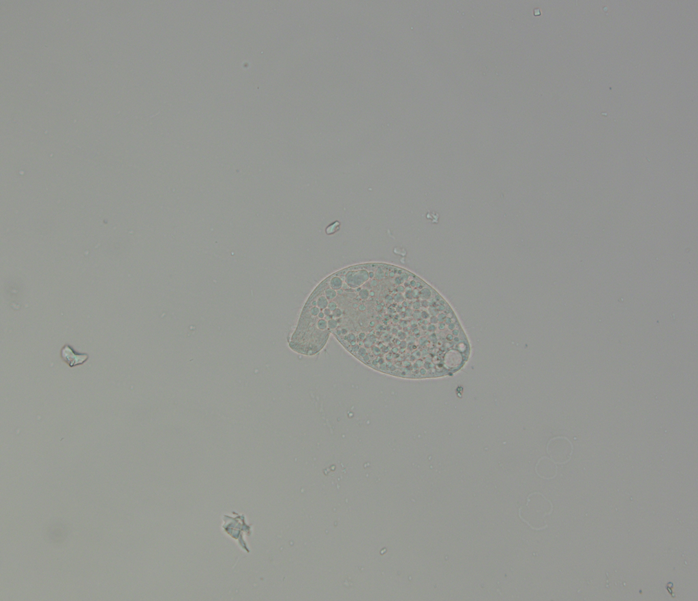


*Tachysoma pellionellum*


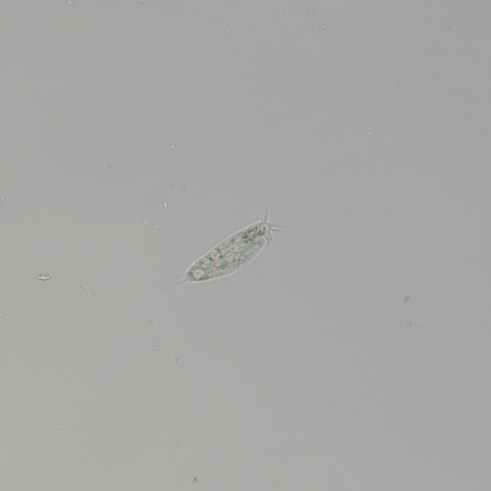


*Stylonchia* sp.1


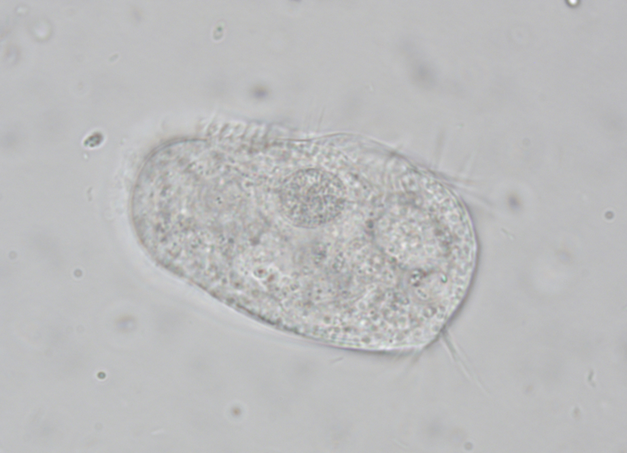


*Trachelophyllum sigmoides*


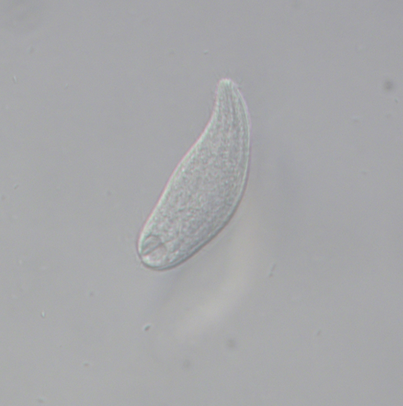


*Urosoma cienkowskii*


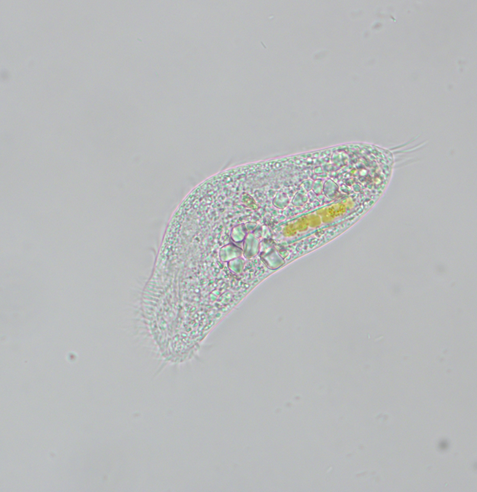


*
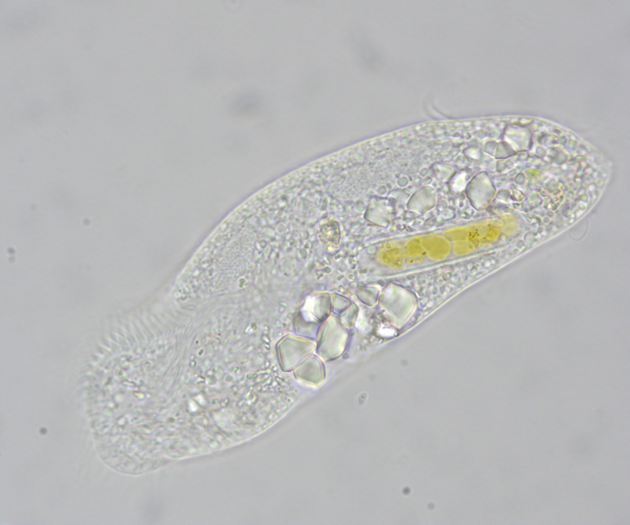
*

*Platyophrya spumacola*


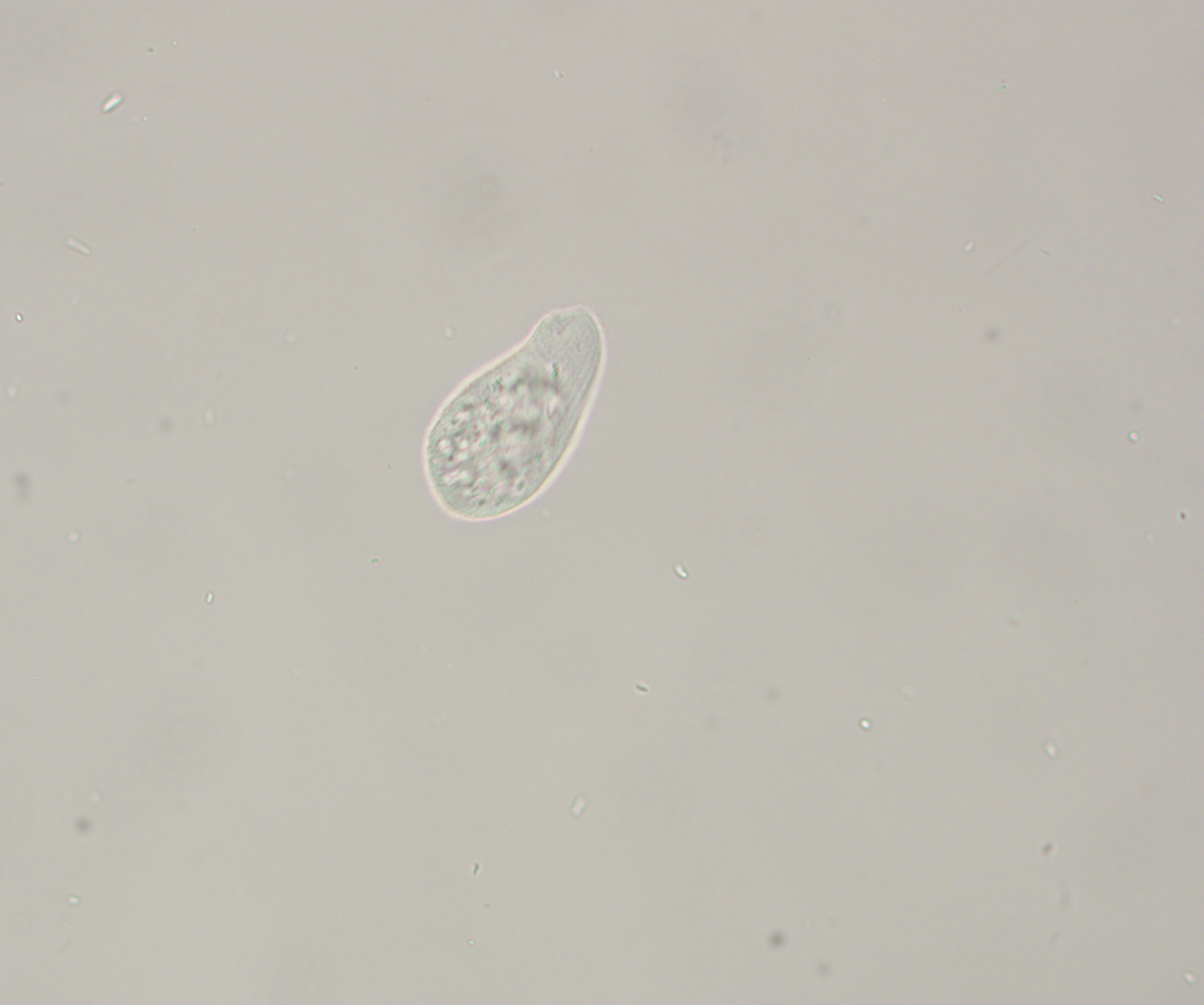

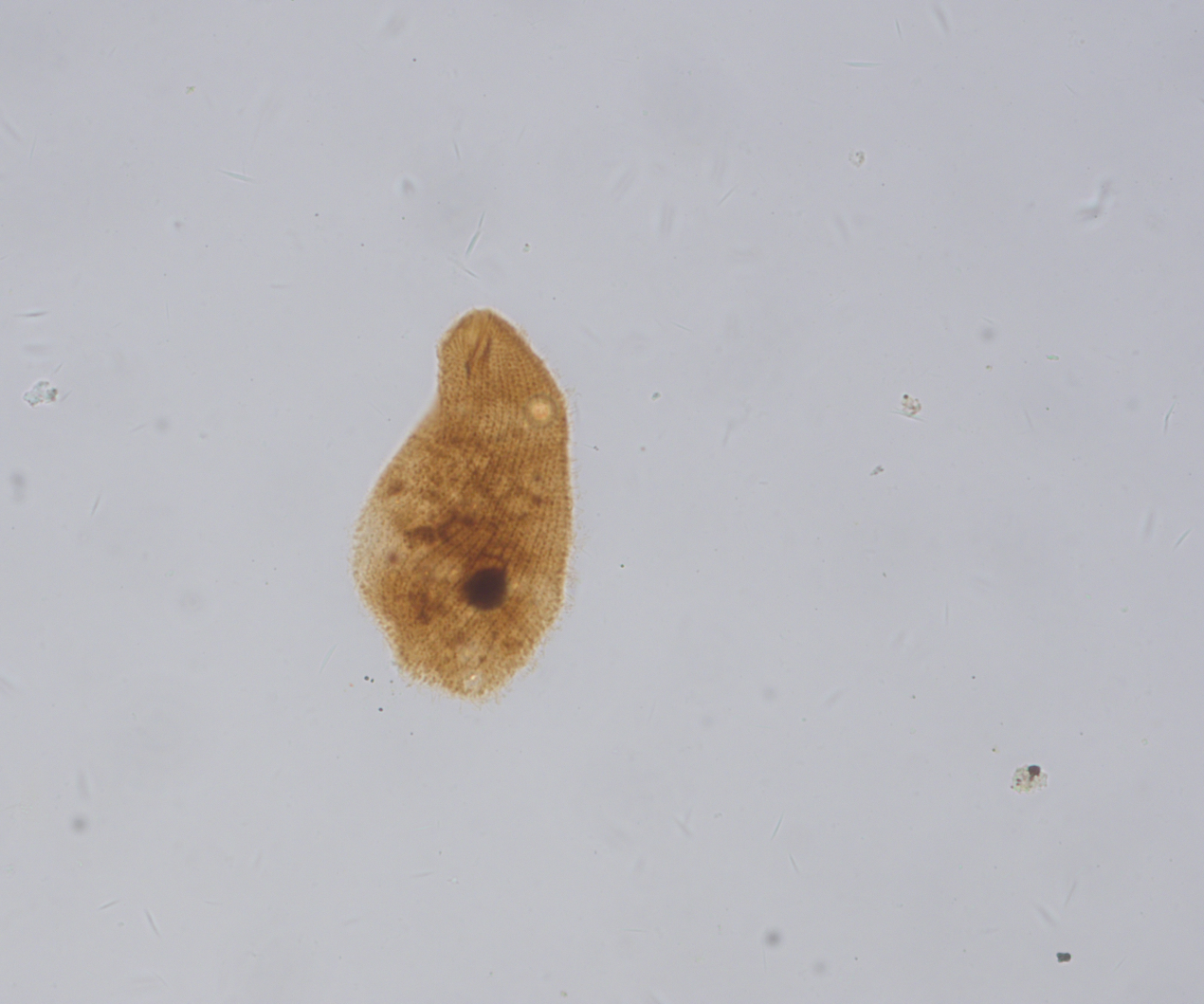


*Holosticha kessleri*


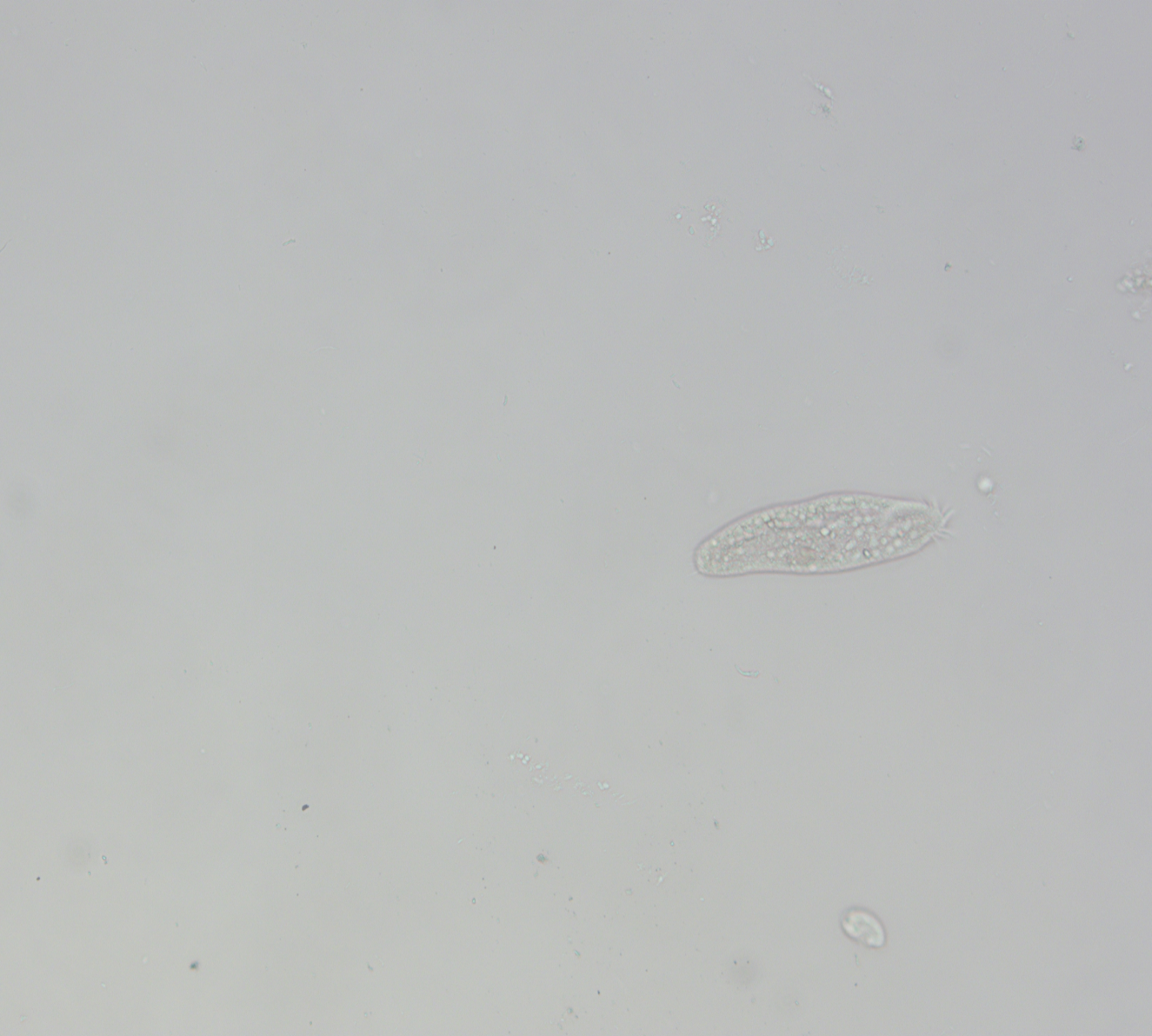

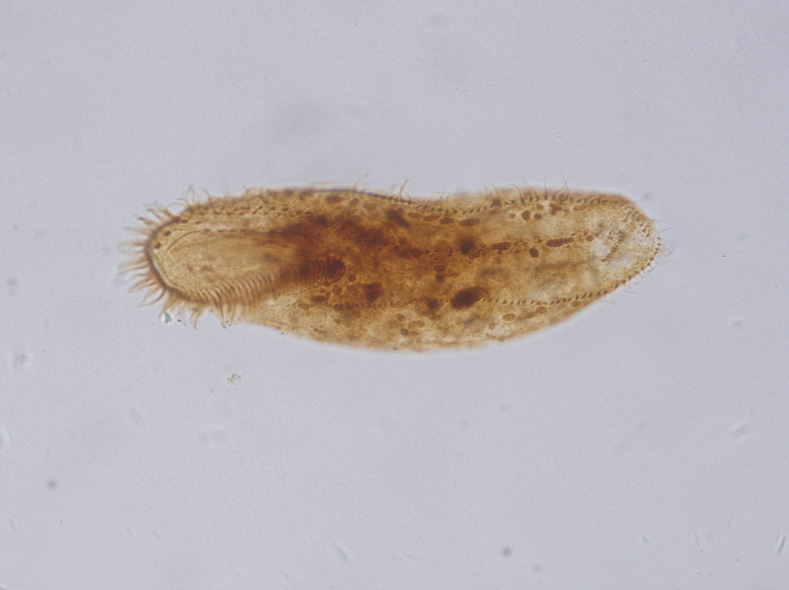


*Blepharisma salinarum*


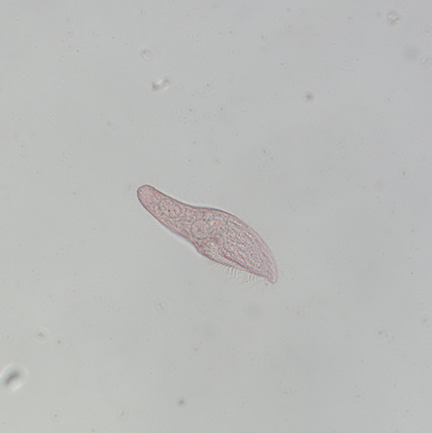


*Sphaerophrya sp.1*


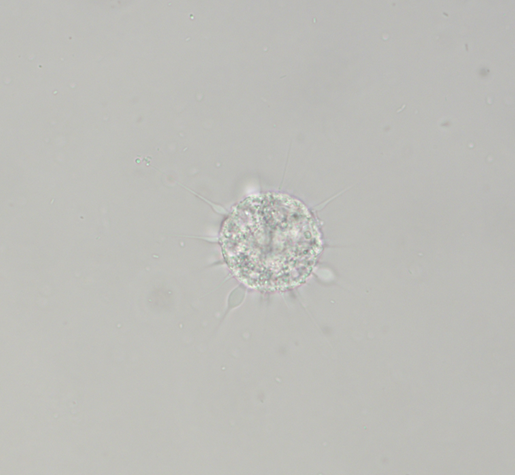


*Cyrtolophosis major*


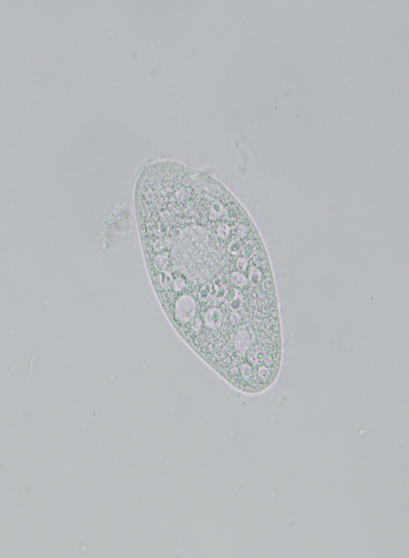


*Paruroleptus caudatus*


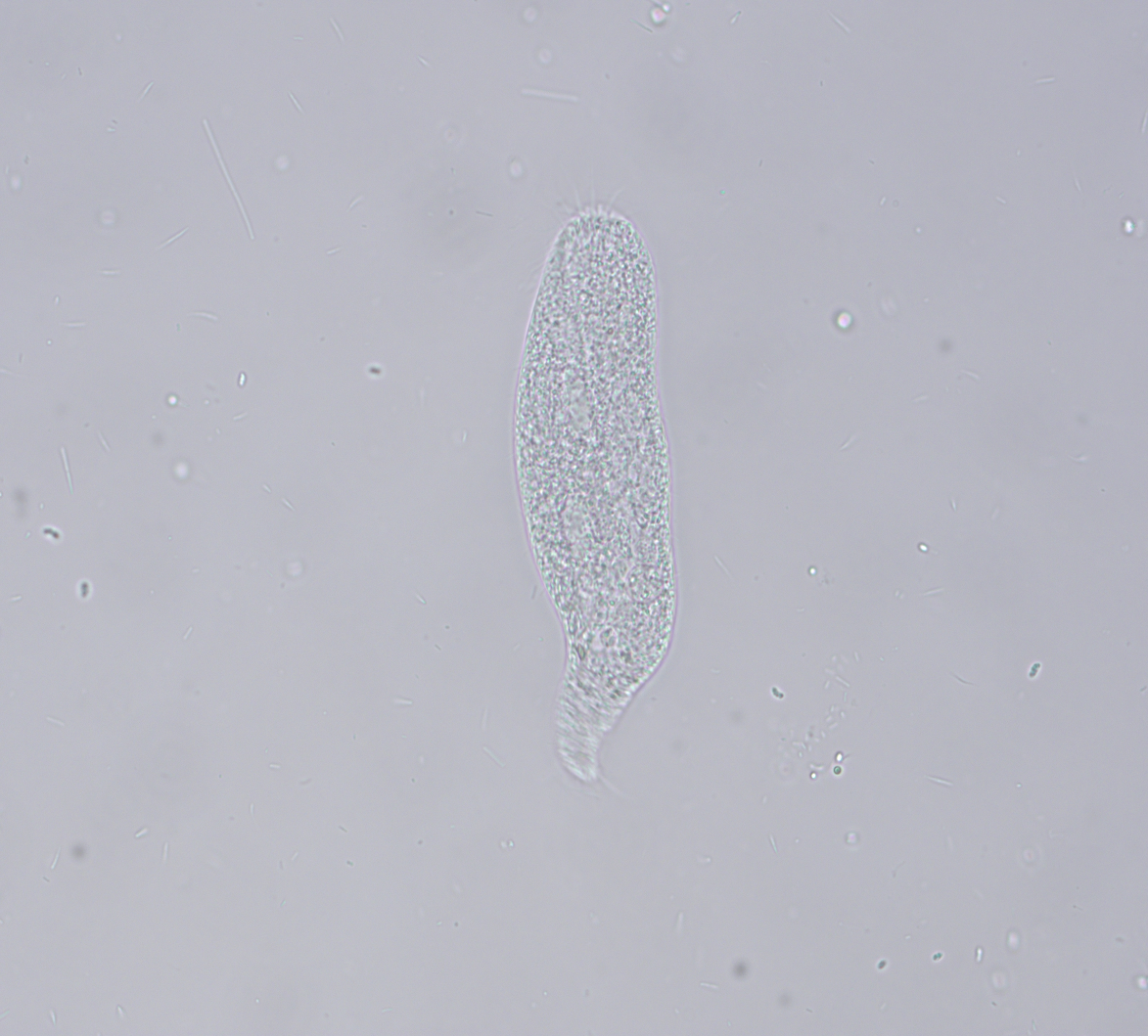

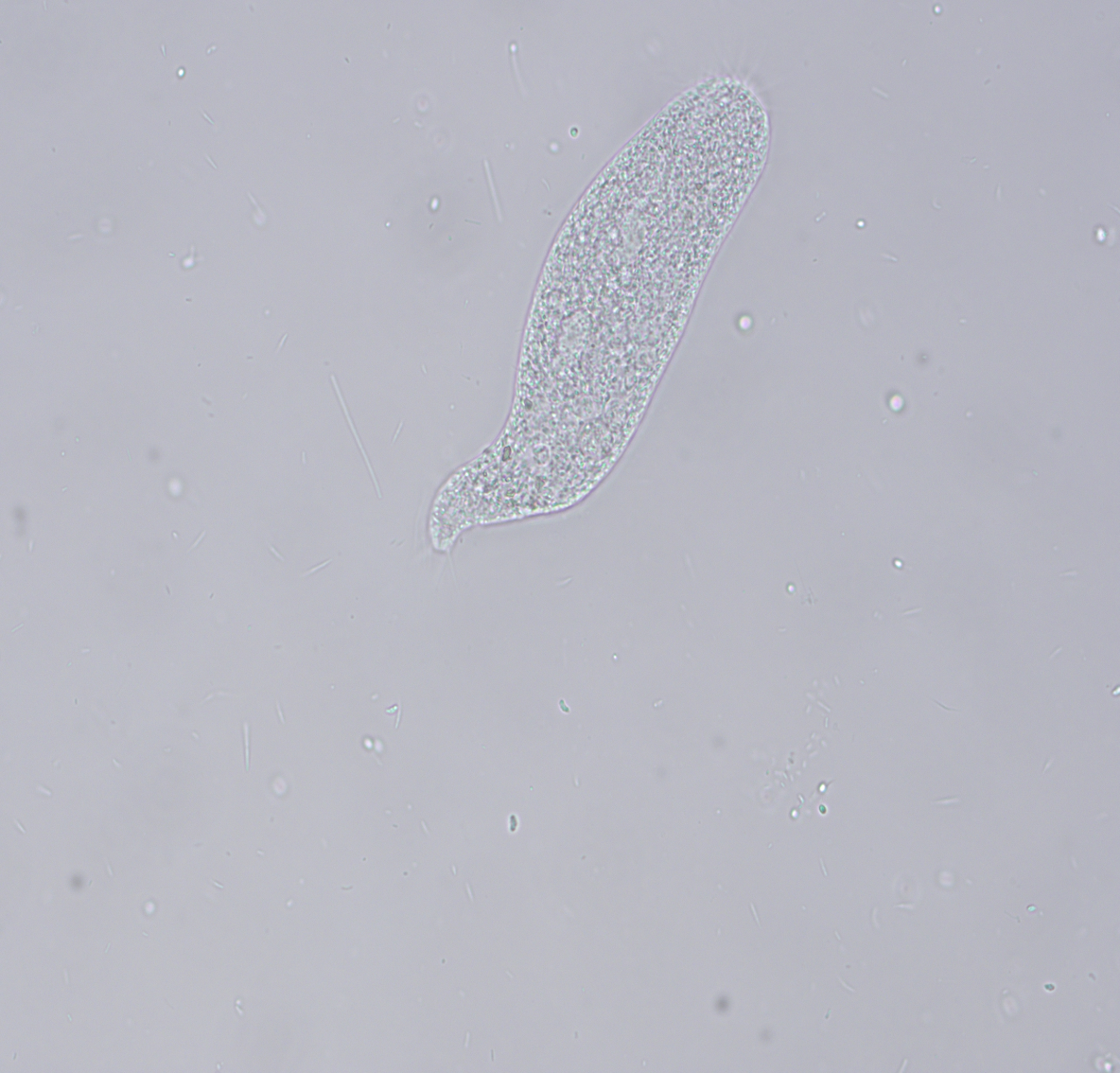


*Opisthotrichum* sp.1


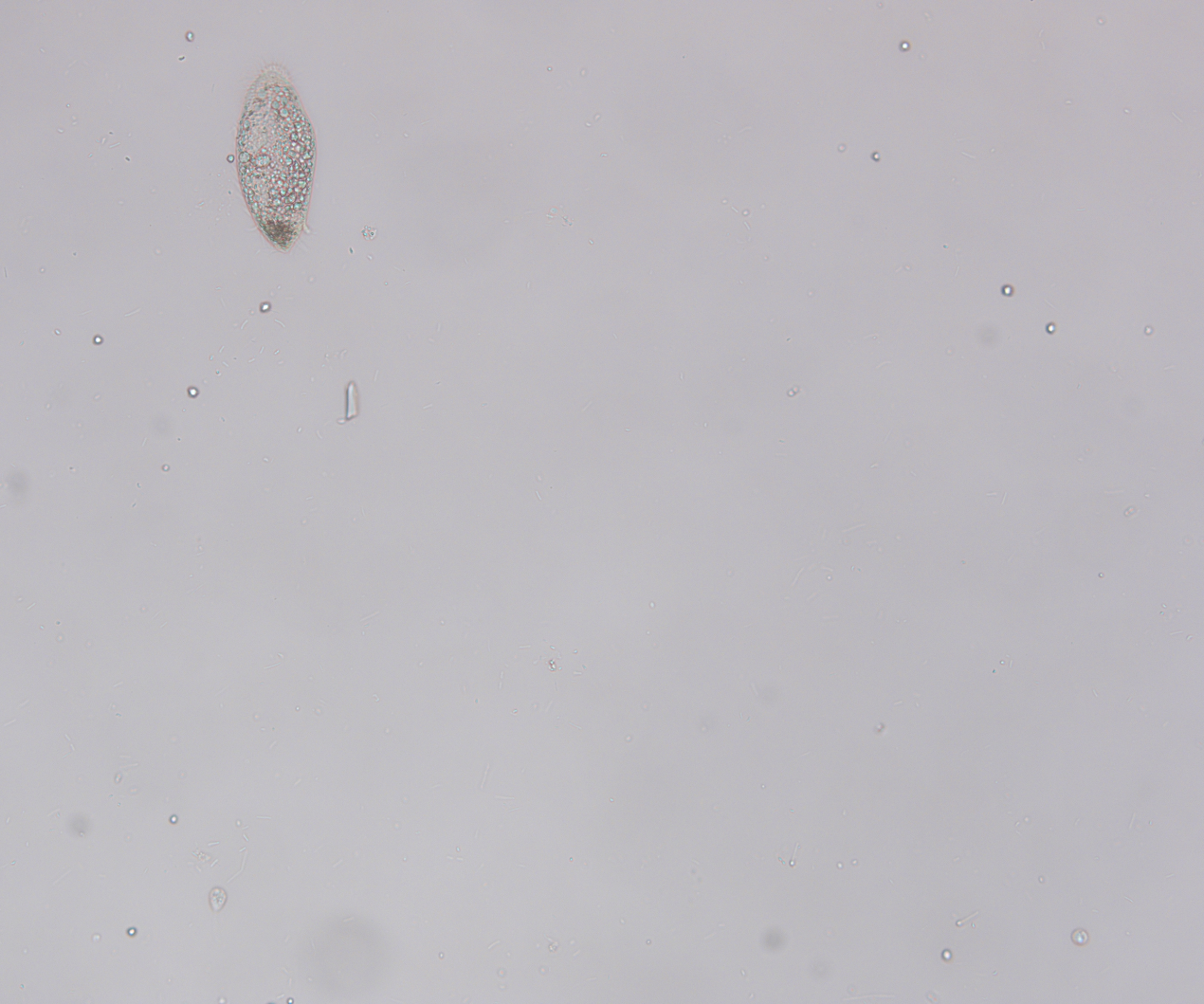


*Litonotus carinatus*


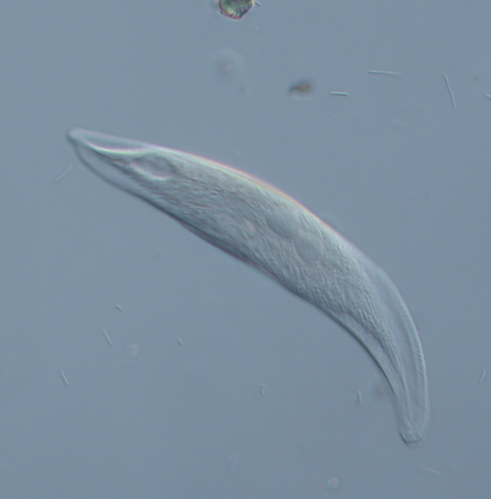

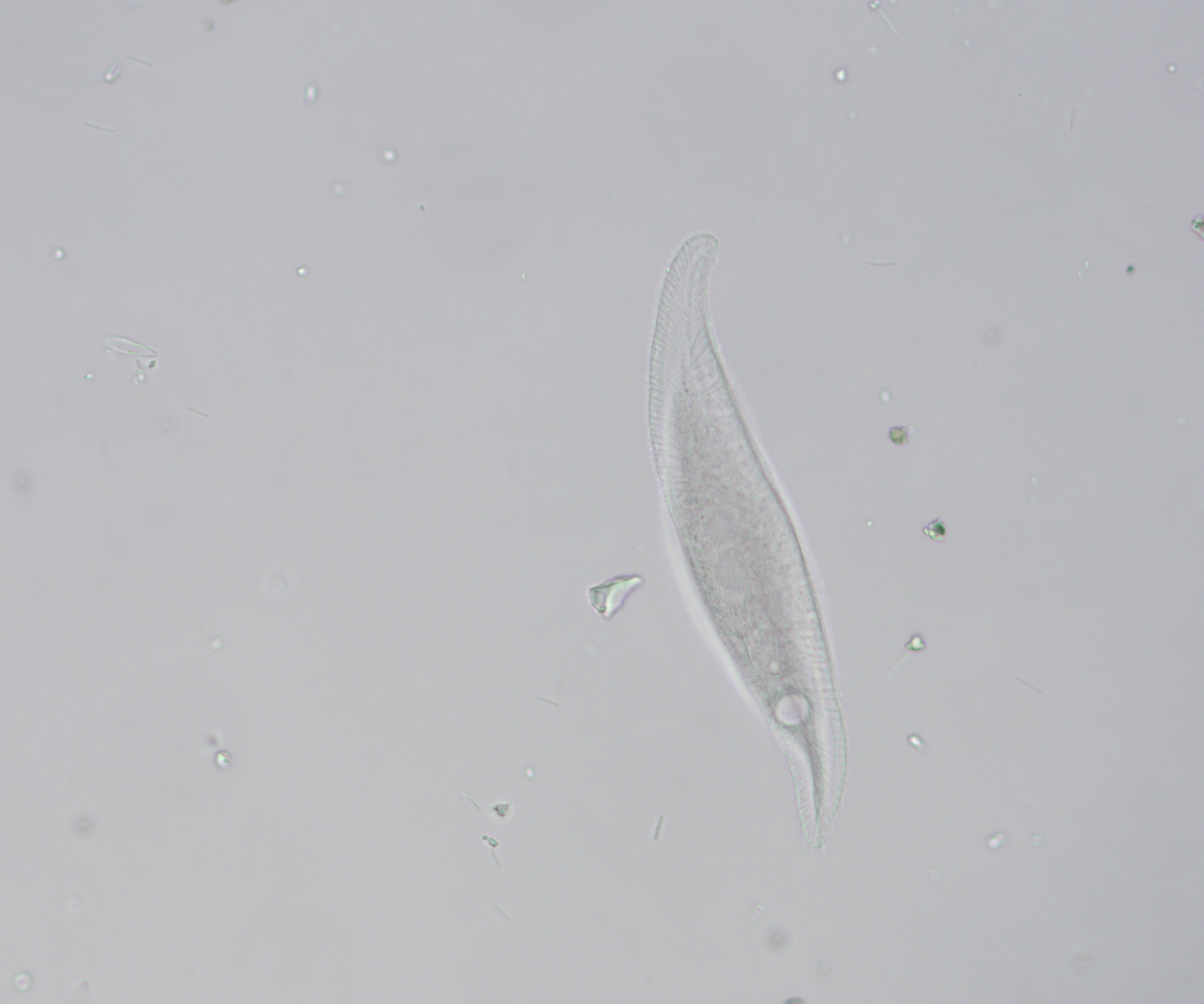


*Opisthotrichum sp.2*


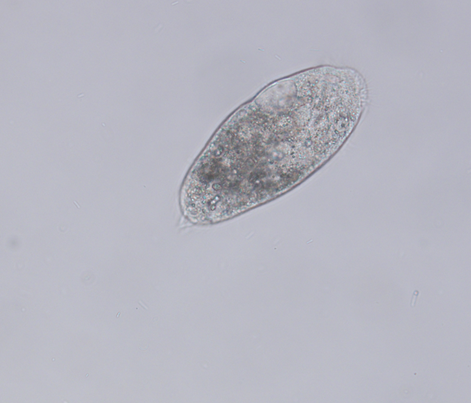


*Holosticha* sp.1


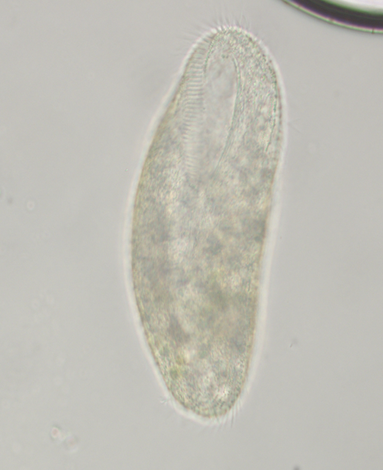


*Dileptus americanus*


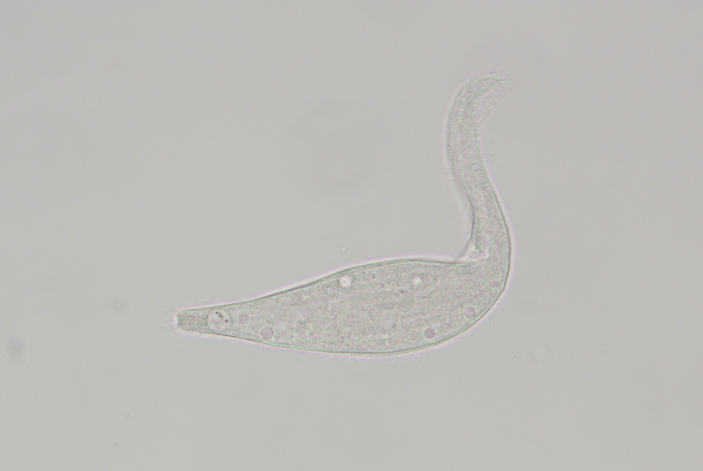


*Vorticella alba*


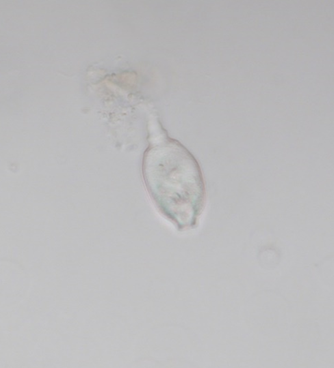

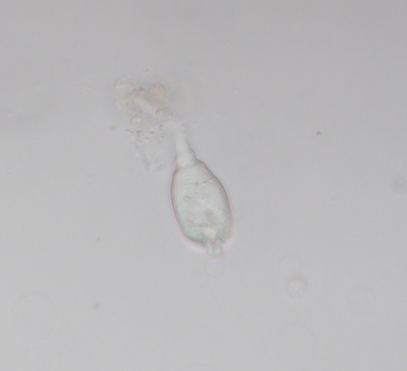


*Vorticella picta*


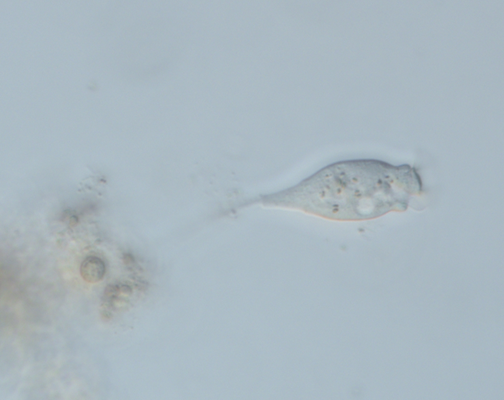

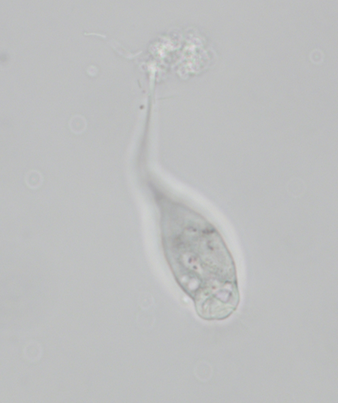


*Vorticella octava*


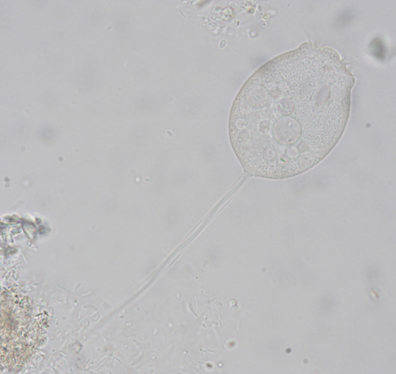

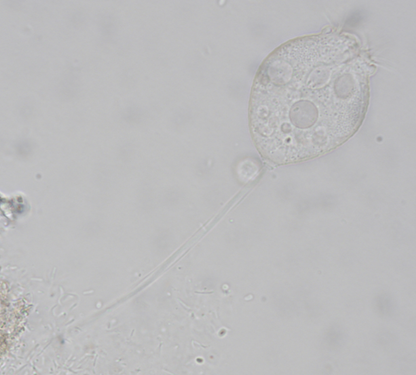


*Paruroleptus musculus*


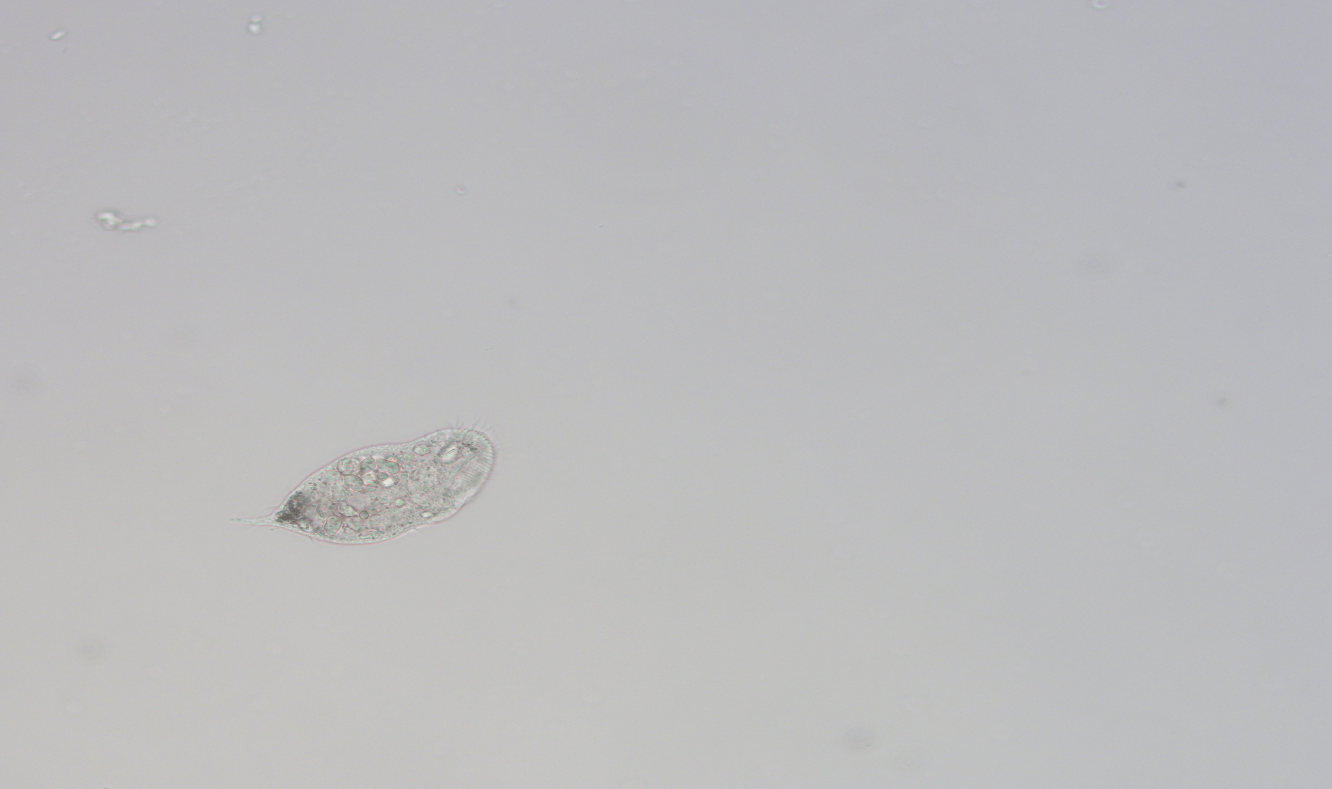


*Leptopharynx eurystoma*


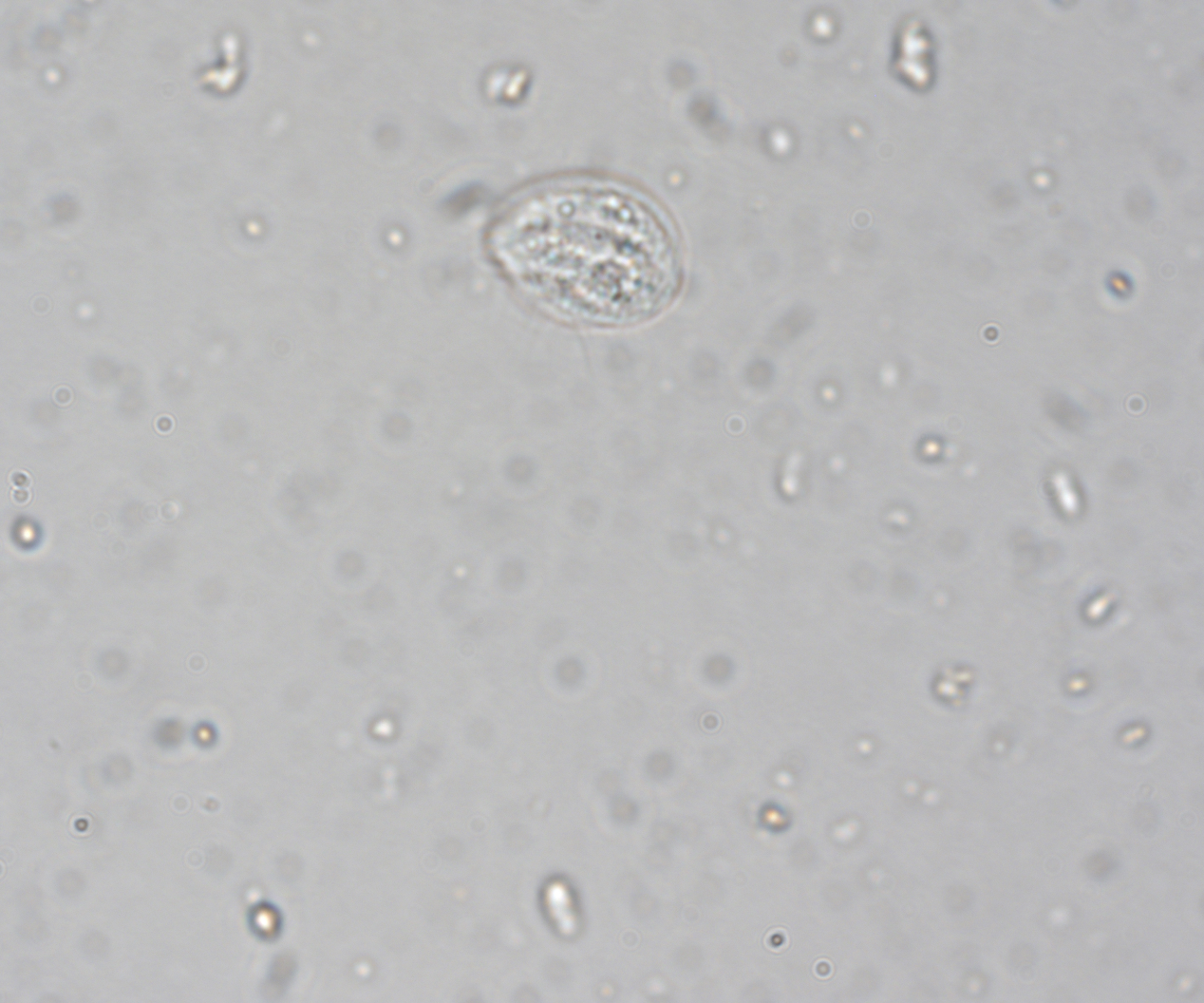


*Spathidium aciculare*


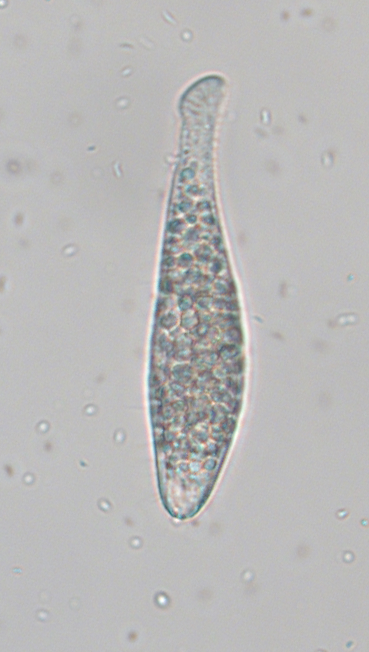

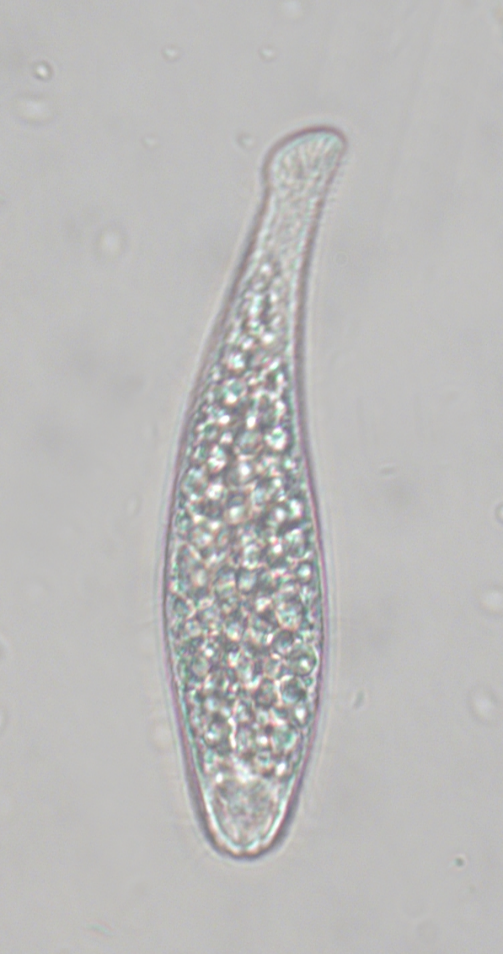


*Spathidium* sp.2


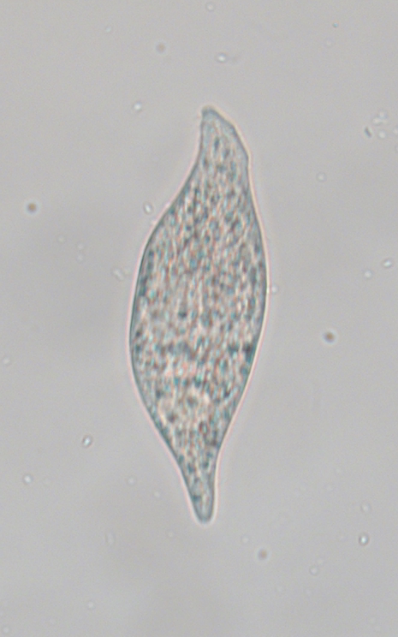

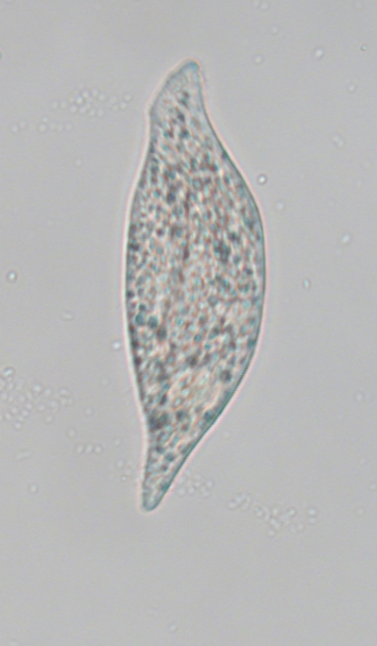


*Oxytricha granulifera*


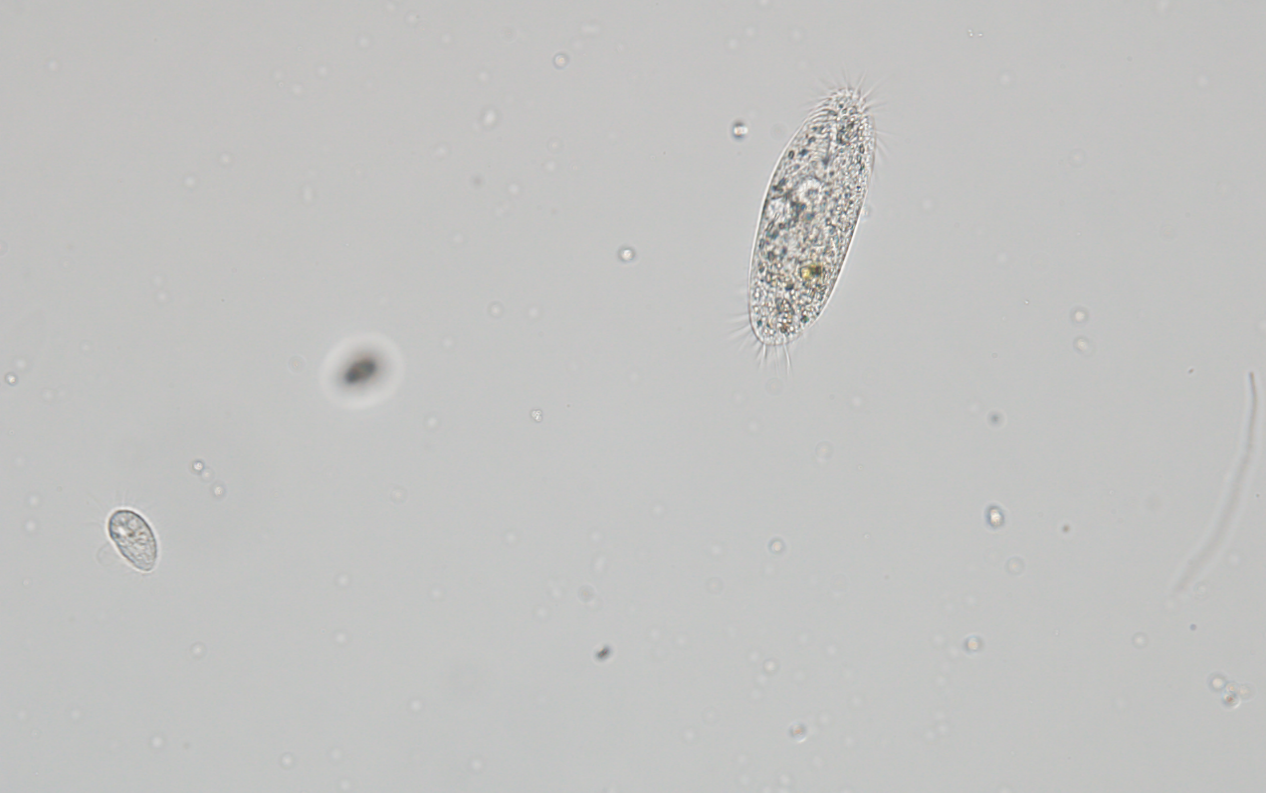

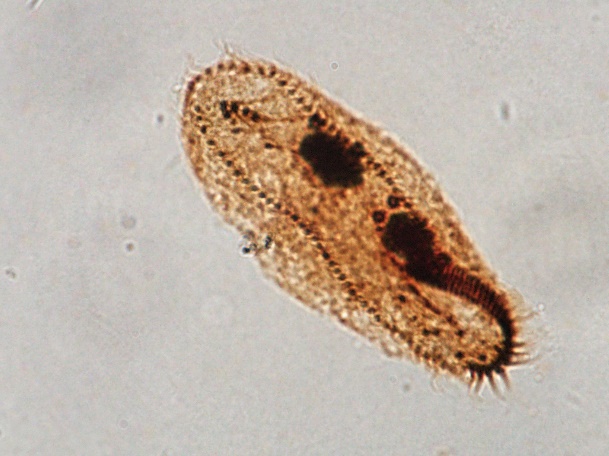


*Spathidium dispar*


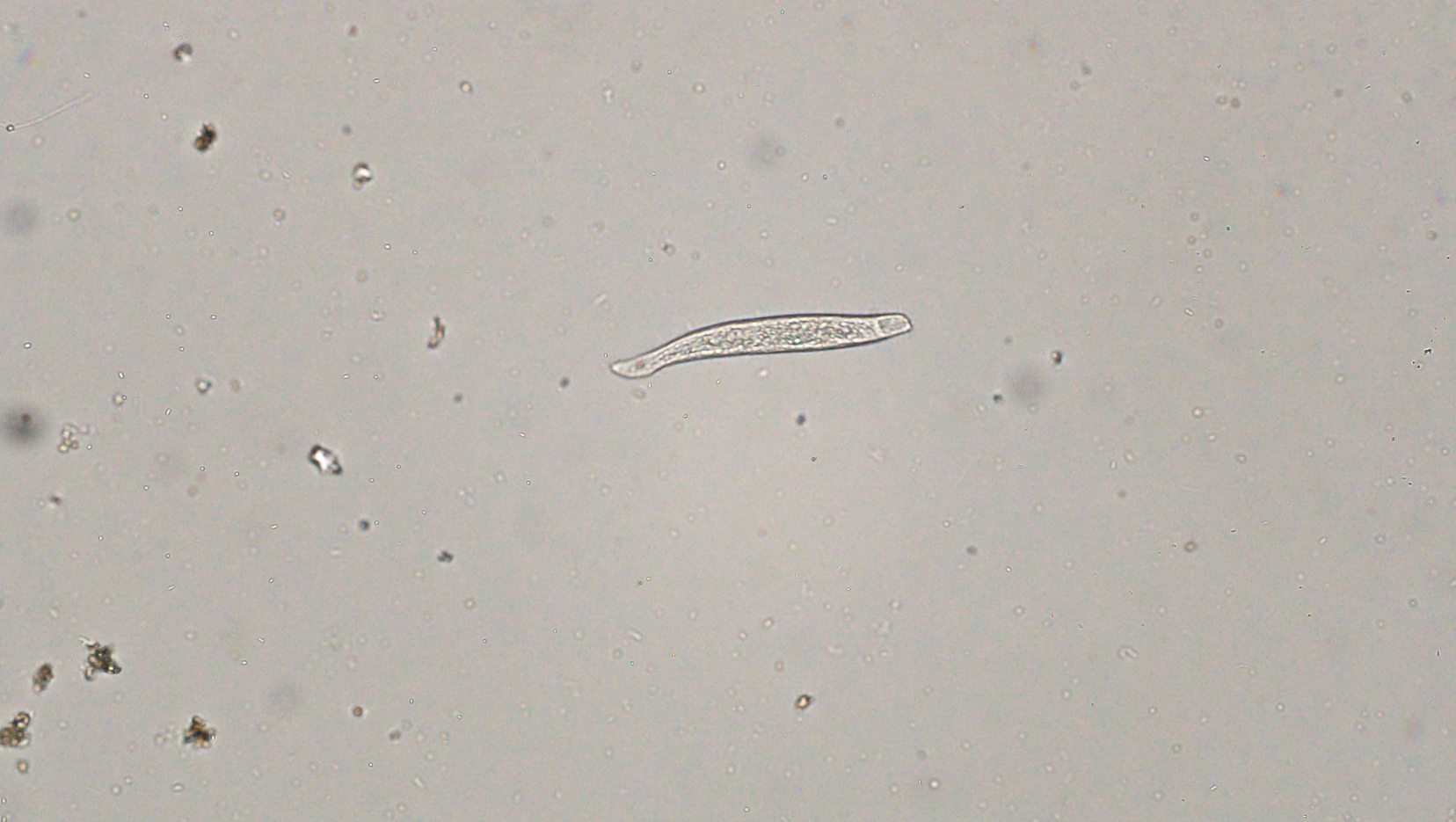

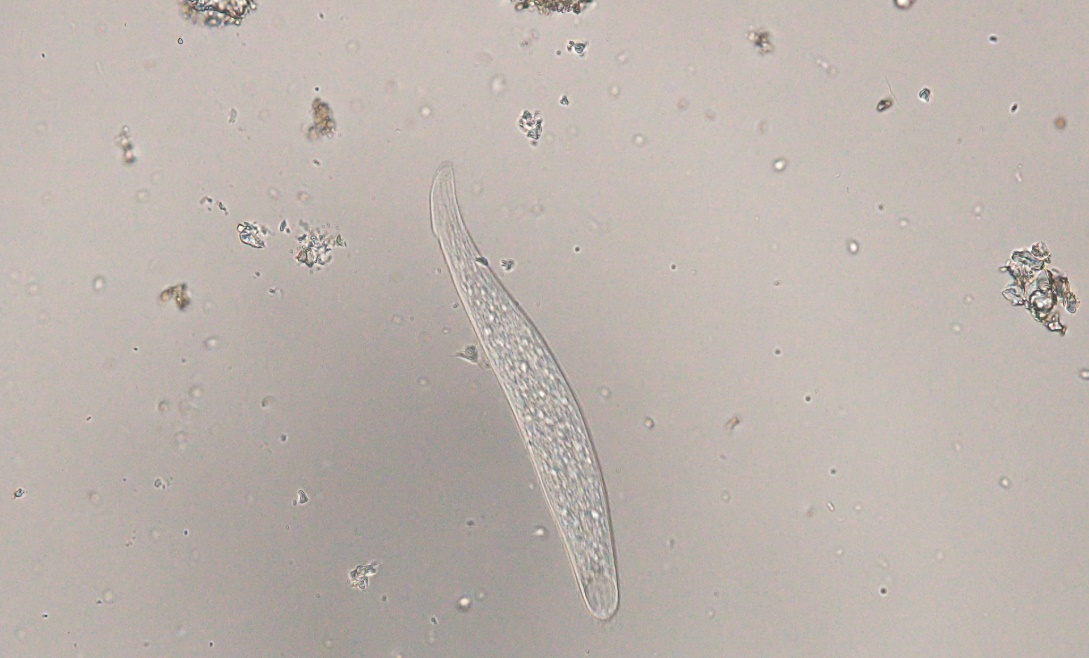


*Enchelys pupa*


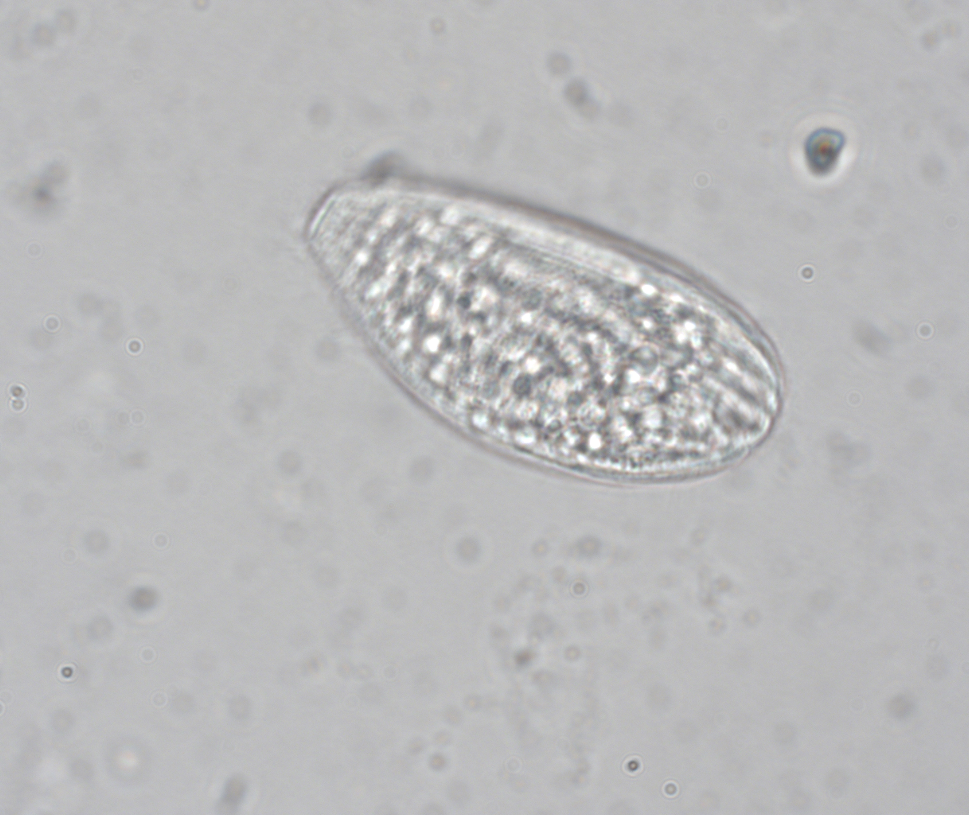

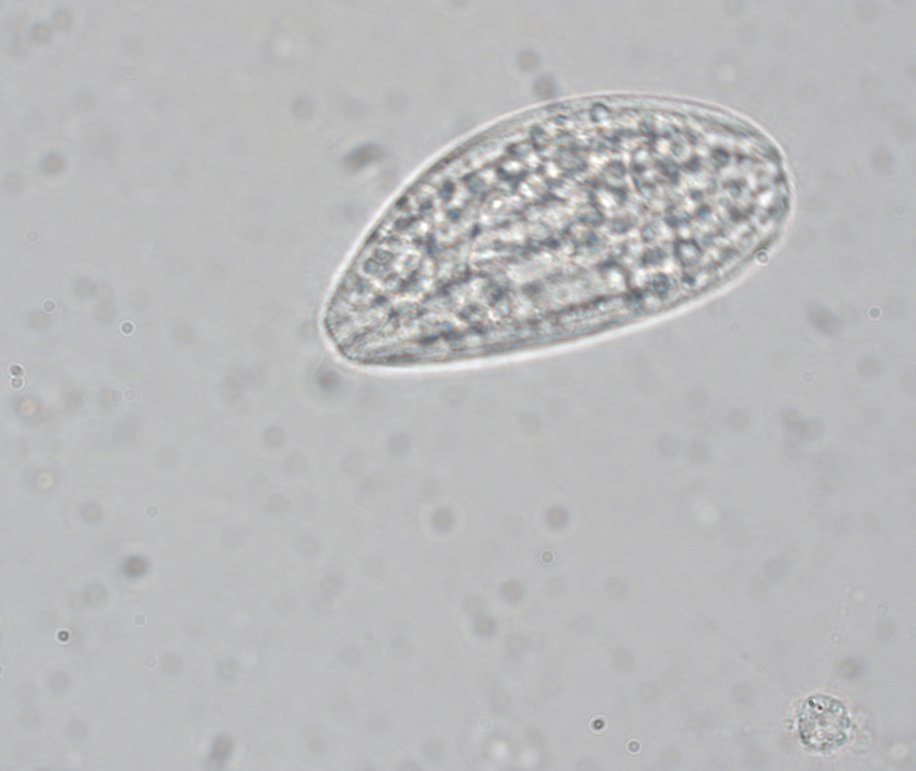


*Uronema nigricans*


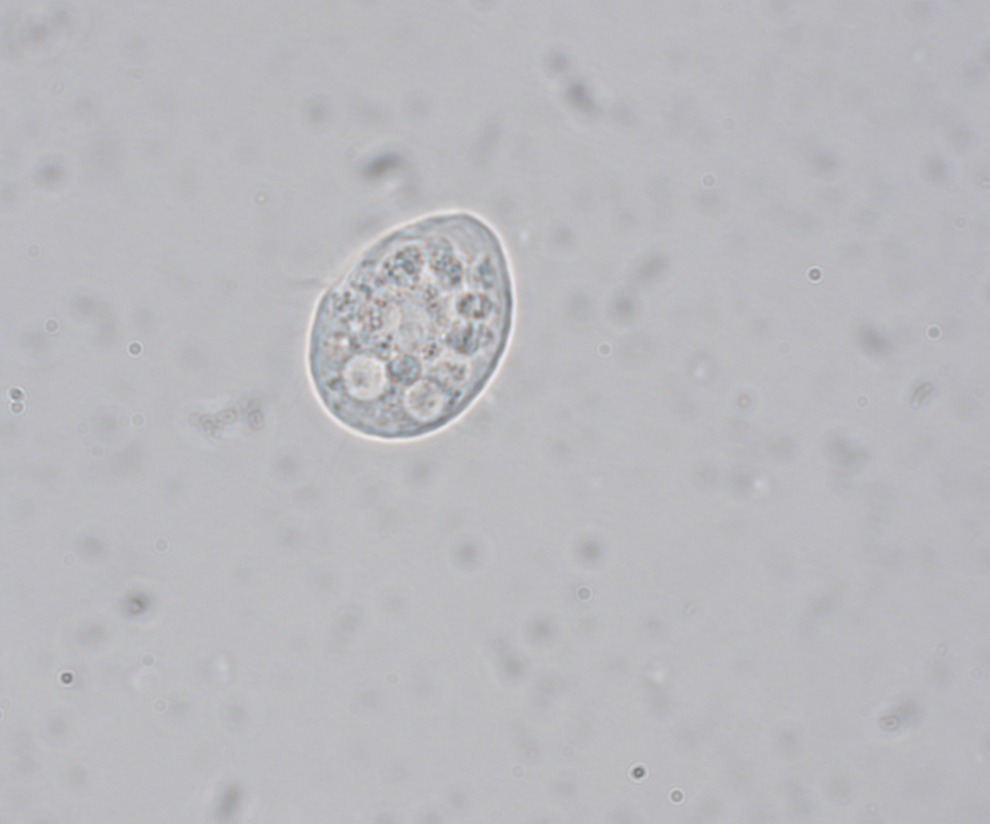


*Spathidium spathula*


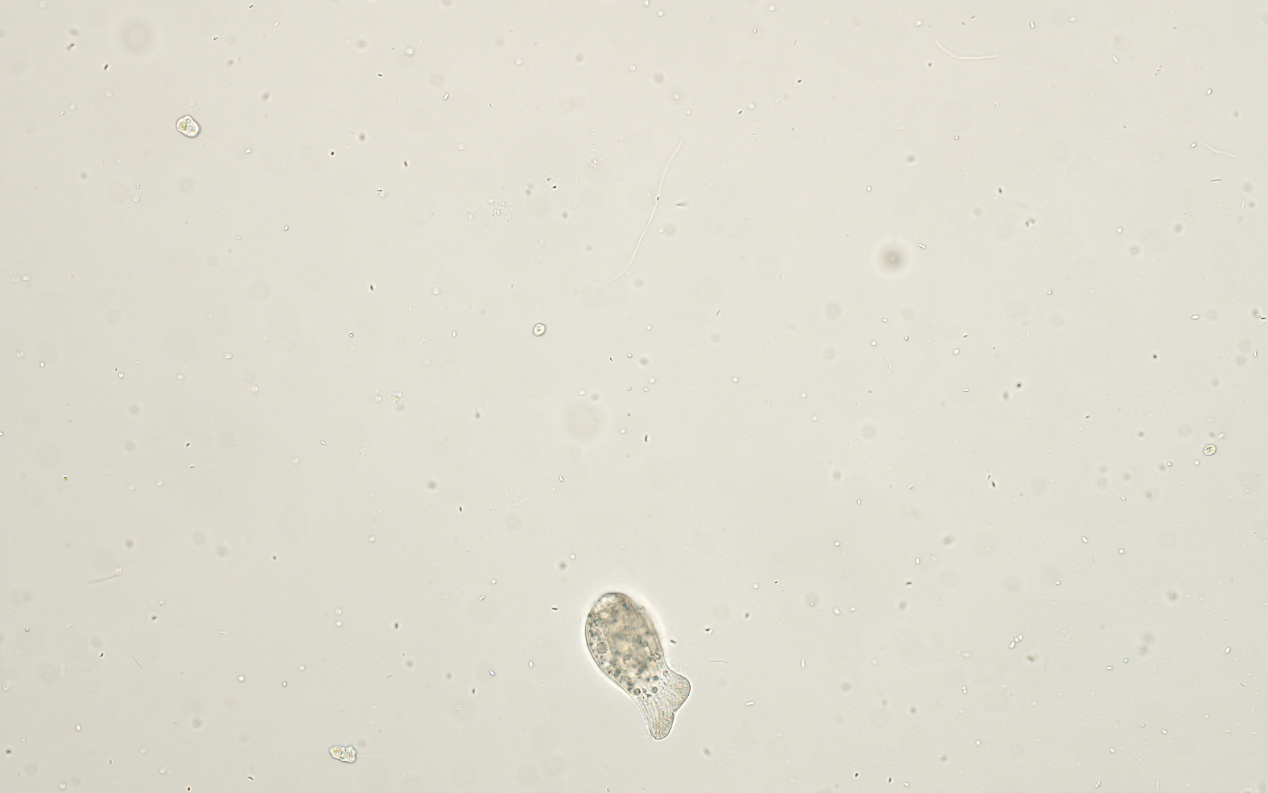

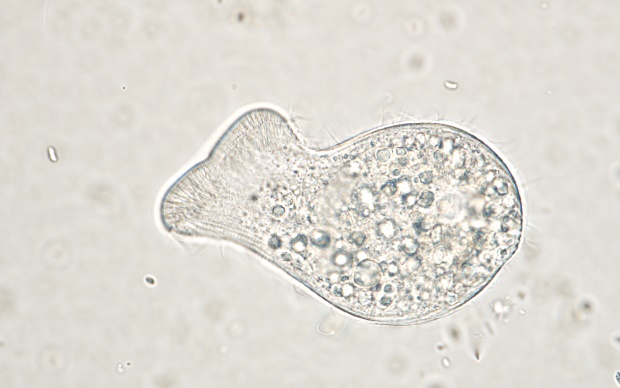


*Pleuronema coronatum*


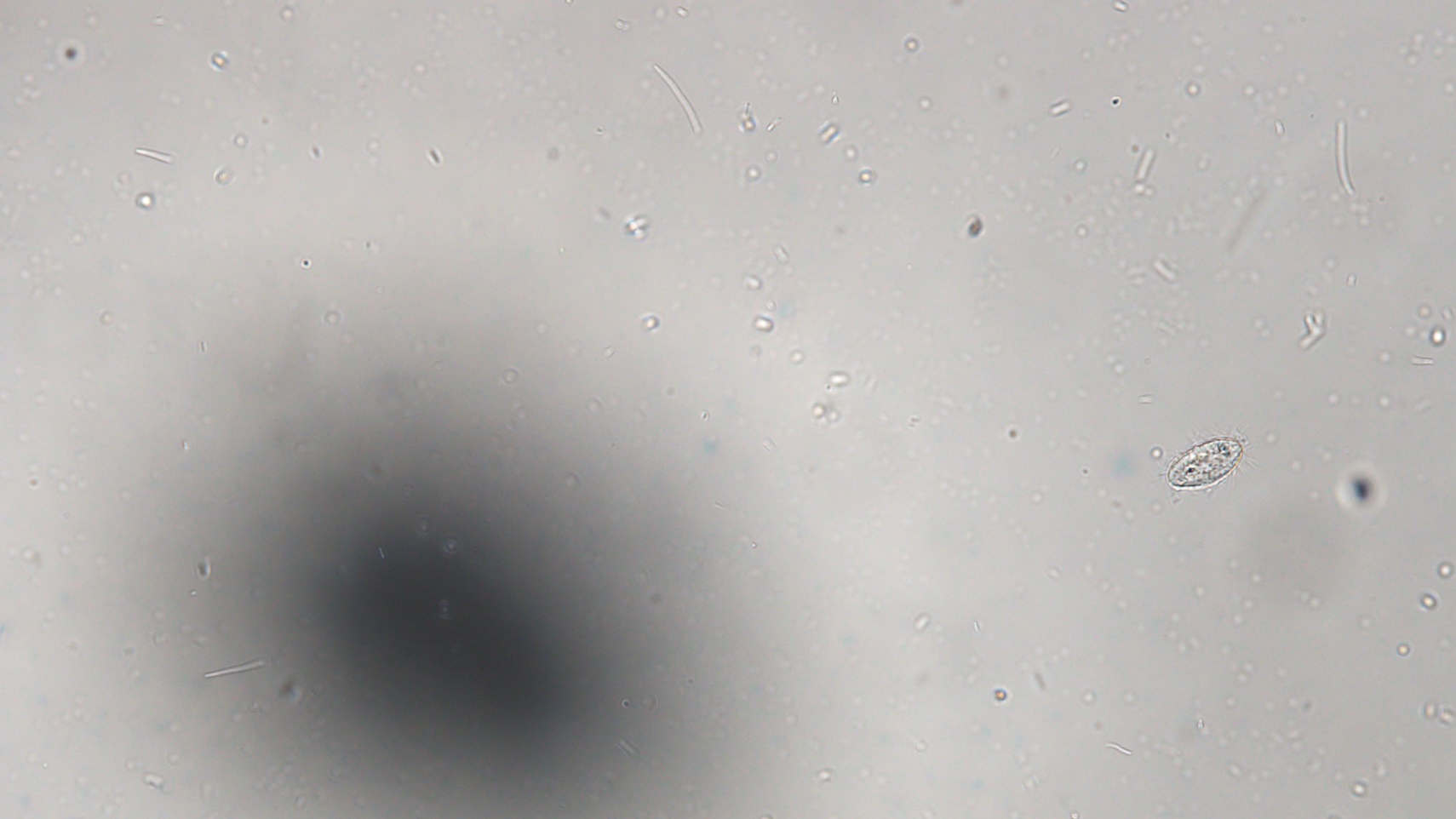

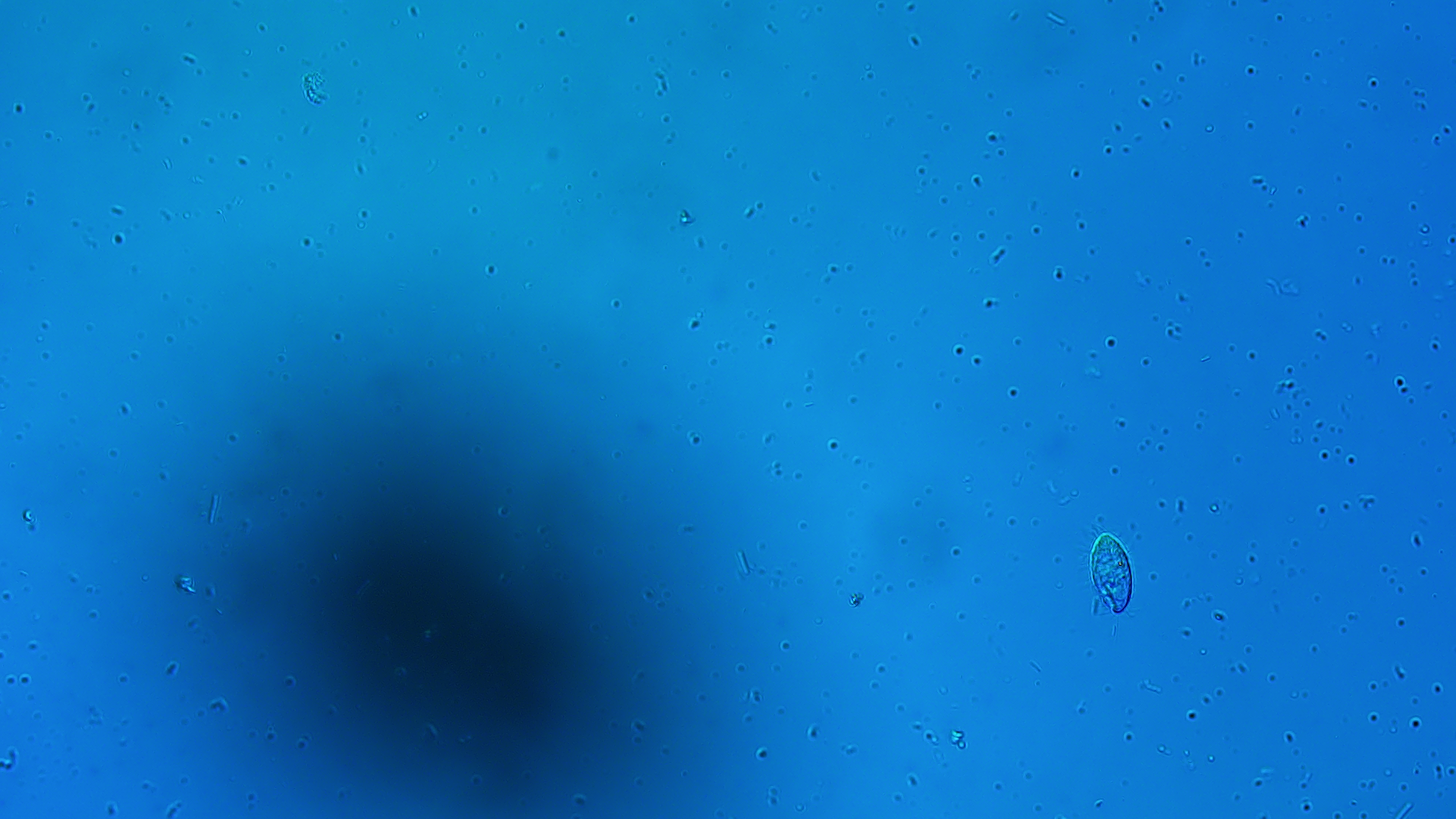


*Acineria uncinata*


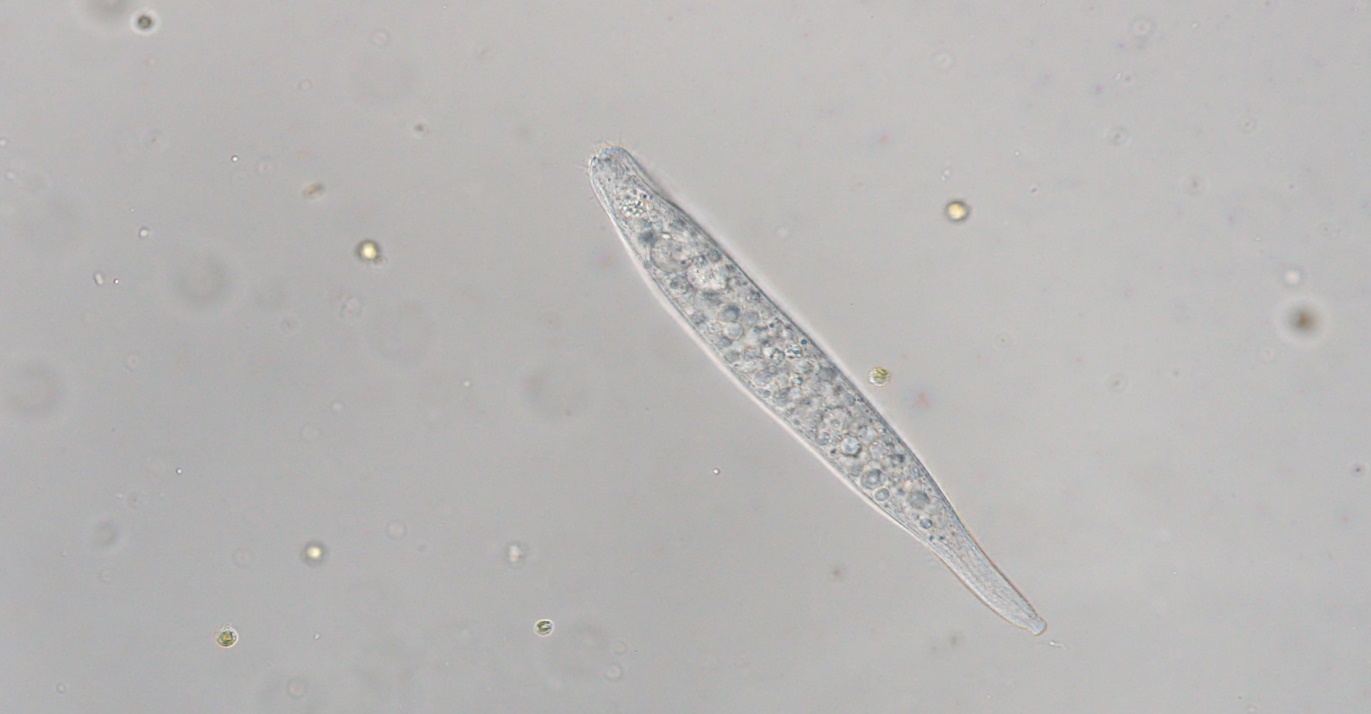


*Sathrophilus oviformis*


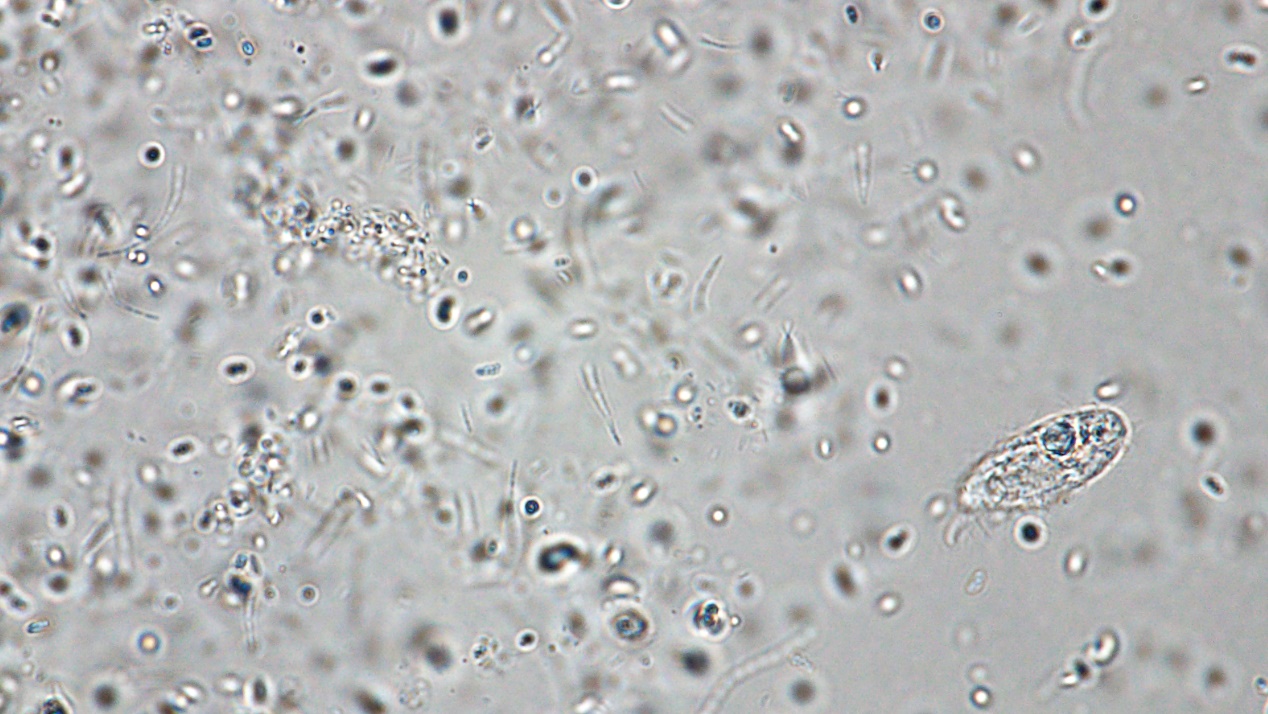

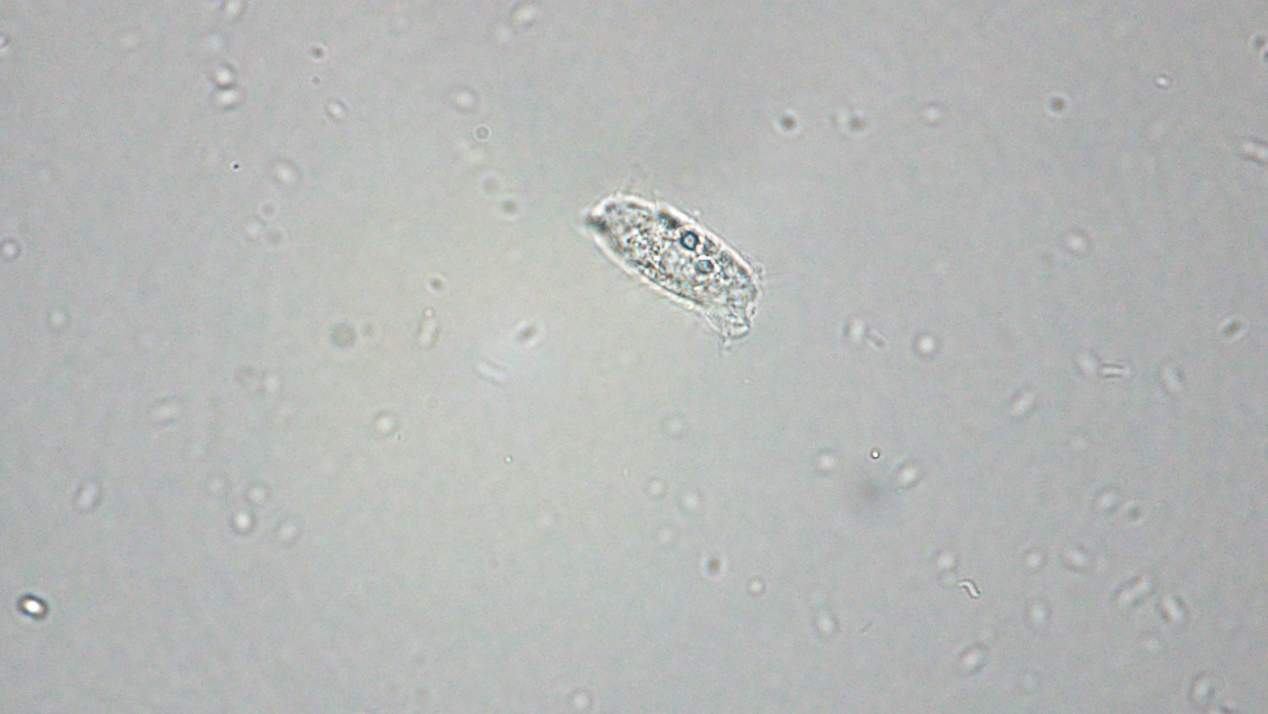


*Spathidium musicola*


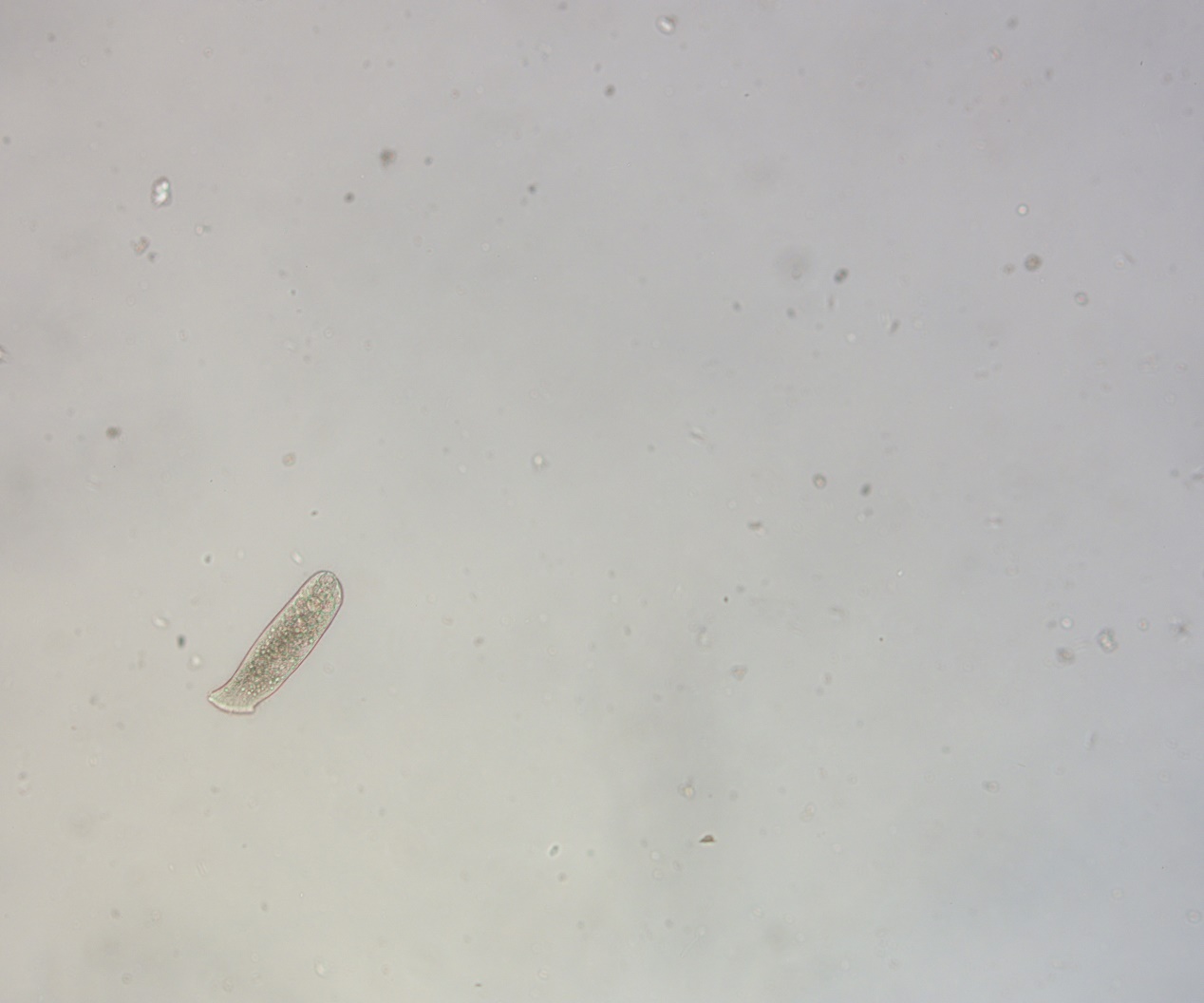


*Vorticella striata*


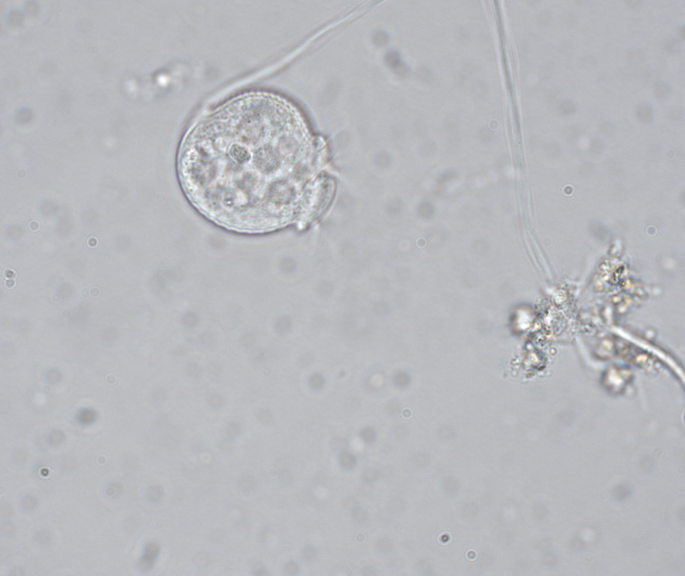

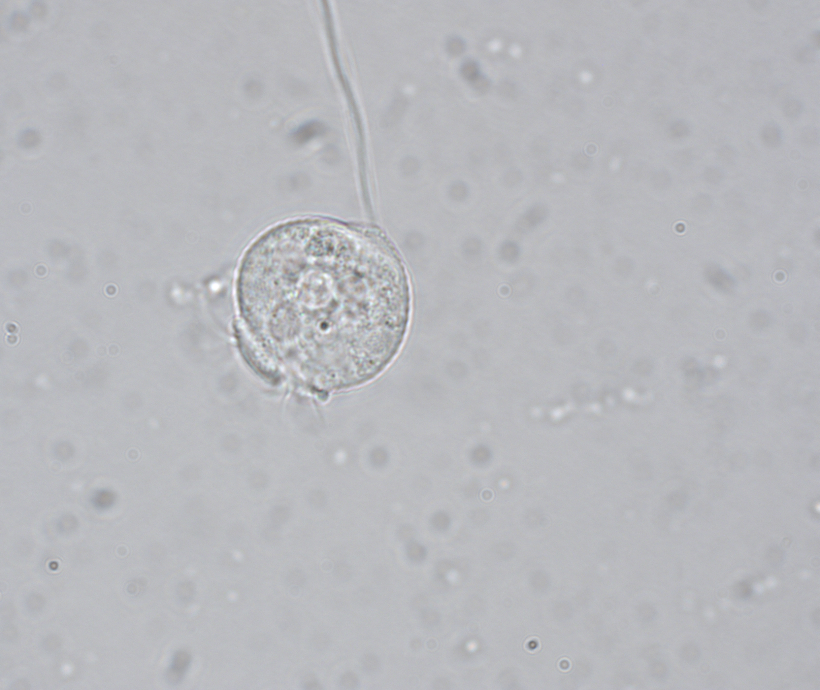


*Vorticella microstoma*


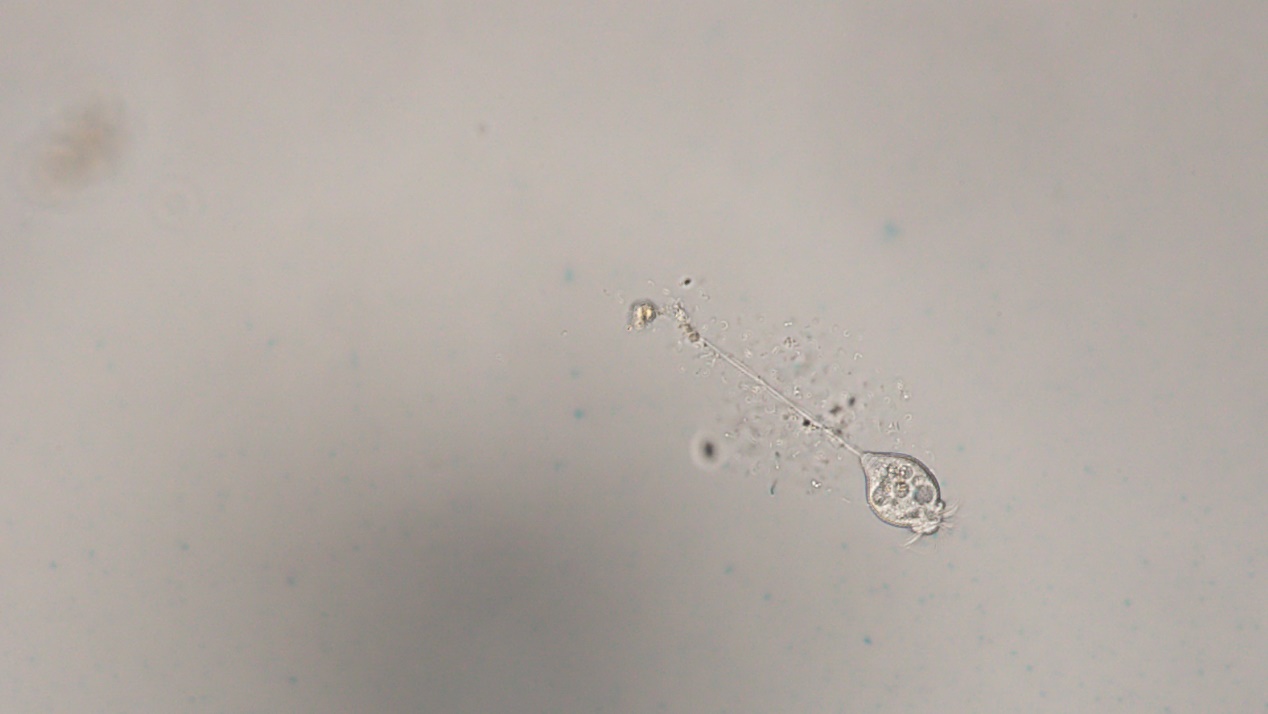


*Plaiyophrya nana*


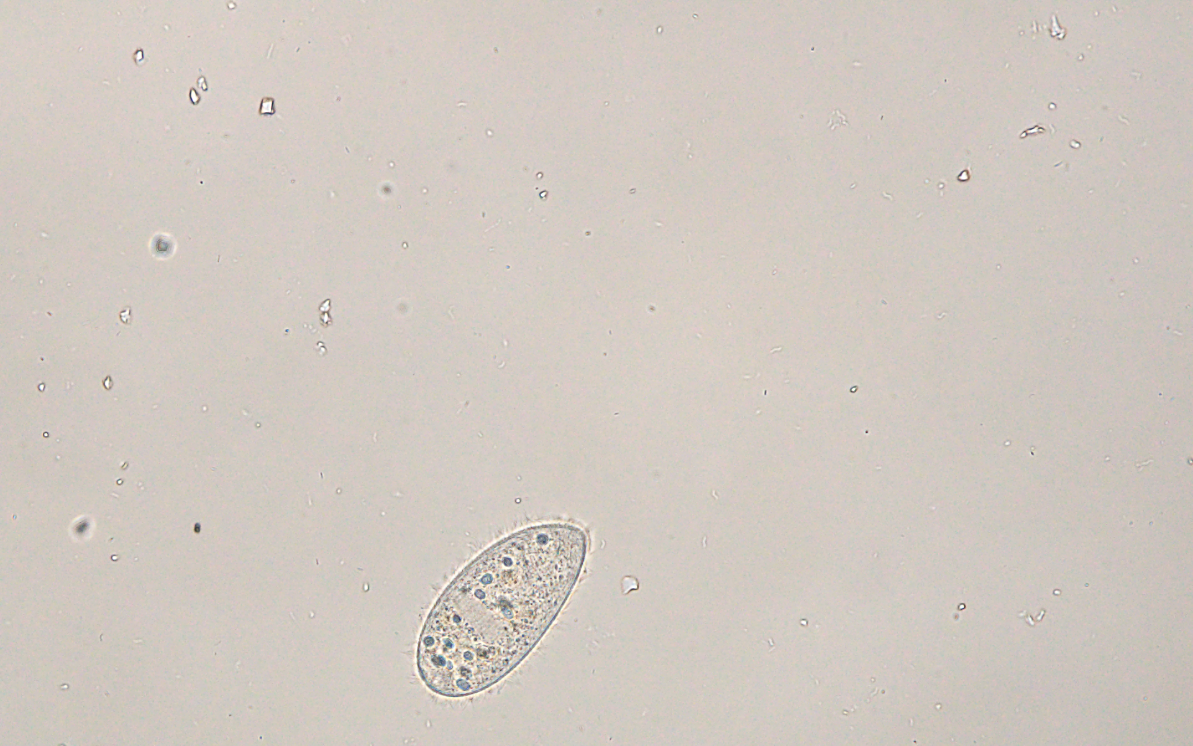

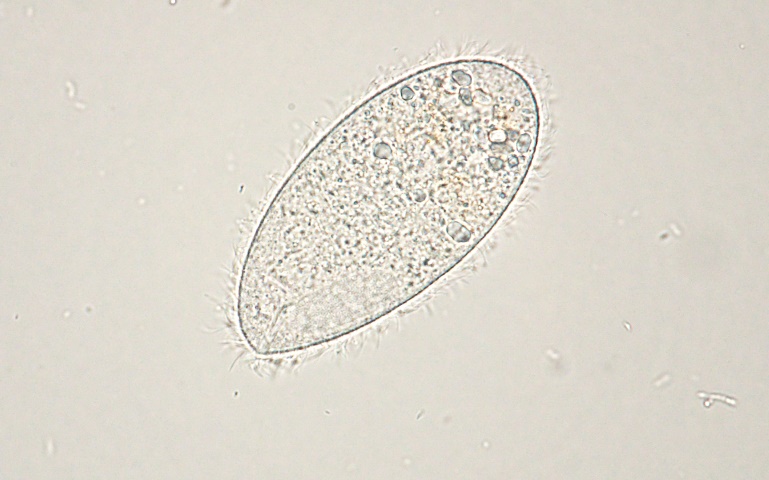


*Stylonychia notophora*


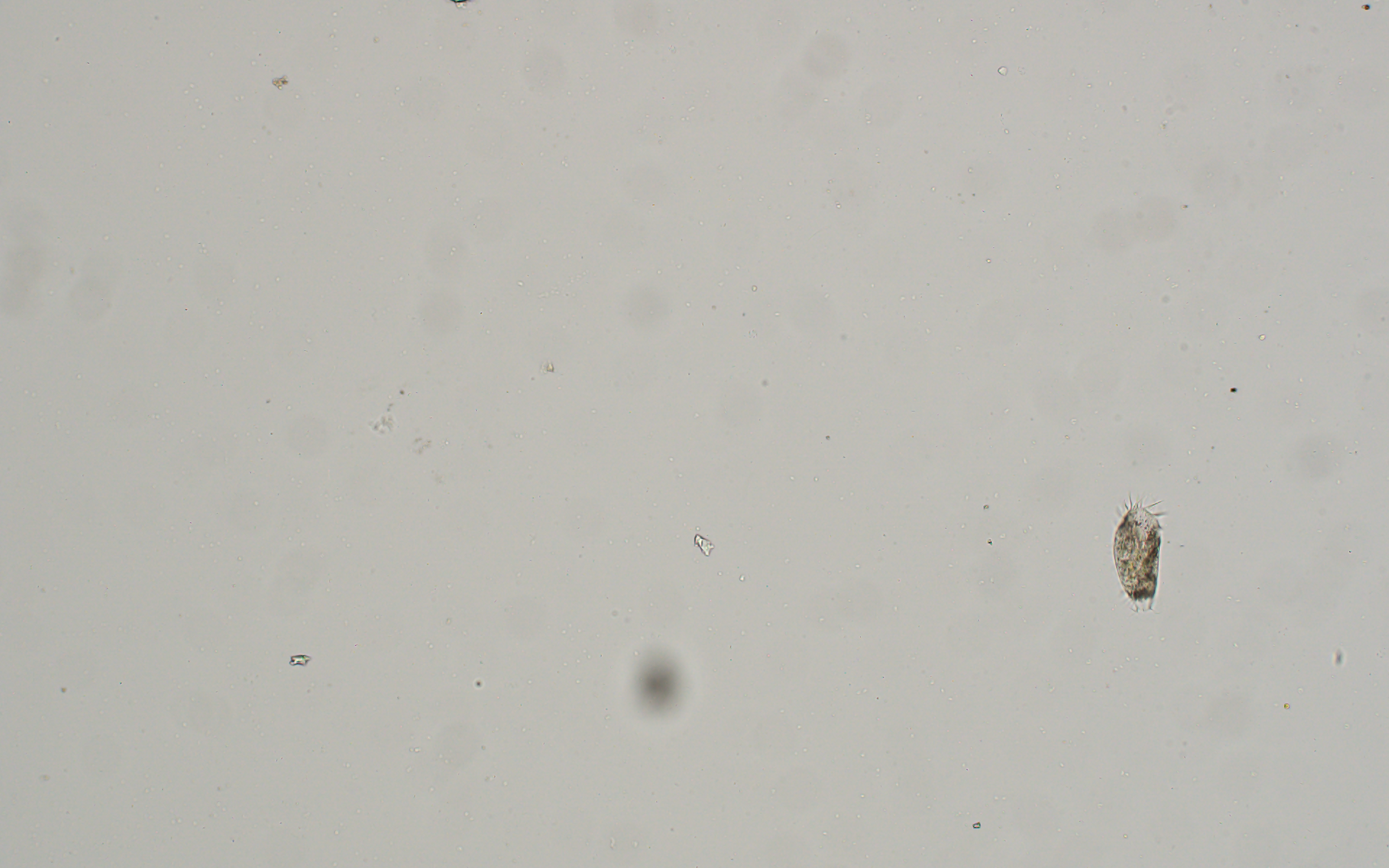

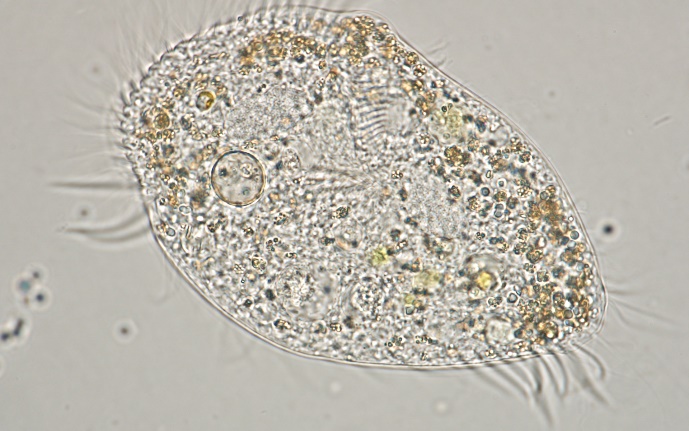


*Oxytricha chlorelligera*


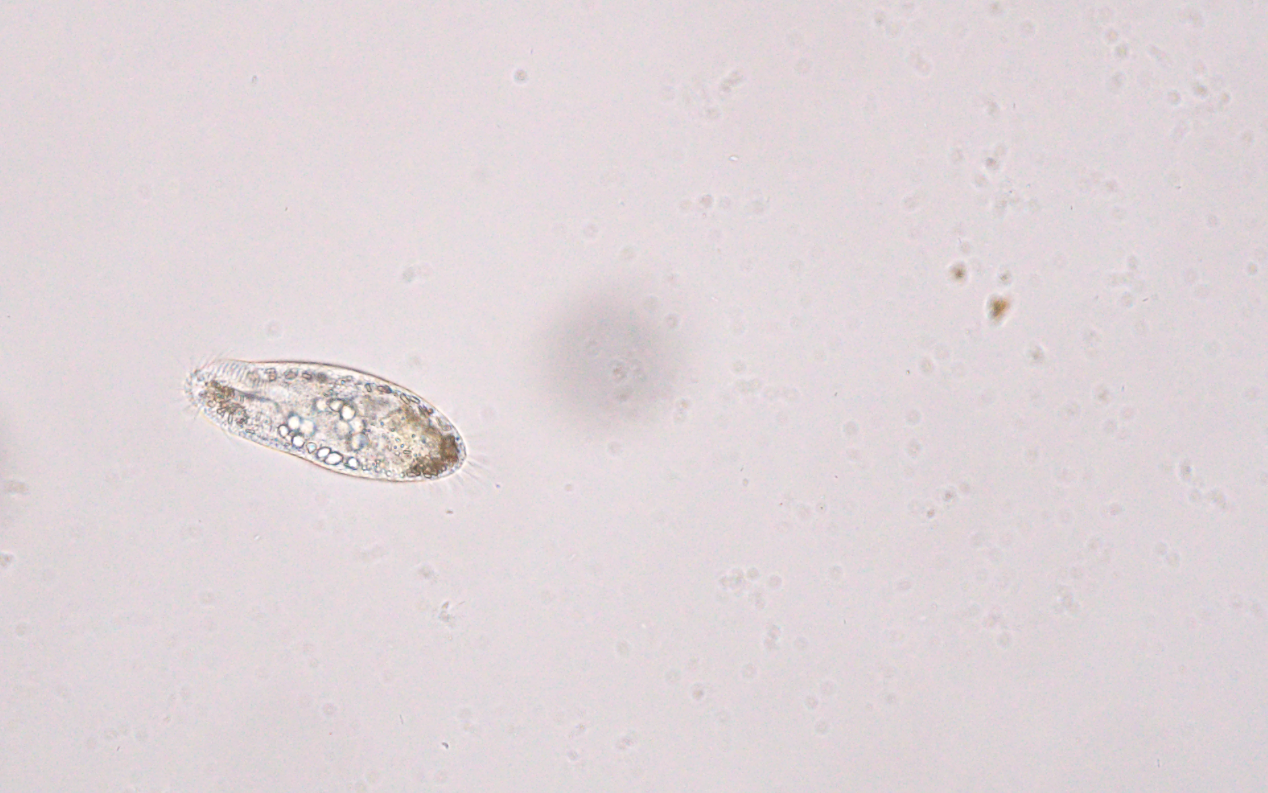


*Tetrahymena pyriformis*


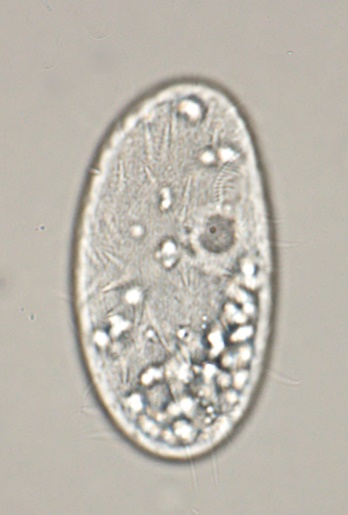


*Dileptus monilatus*


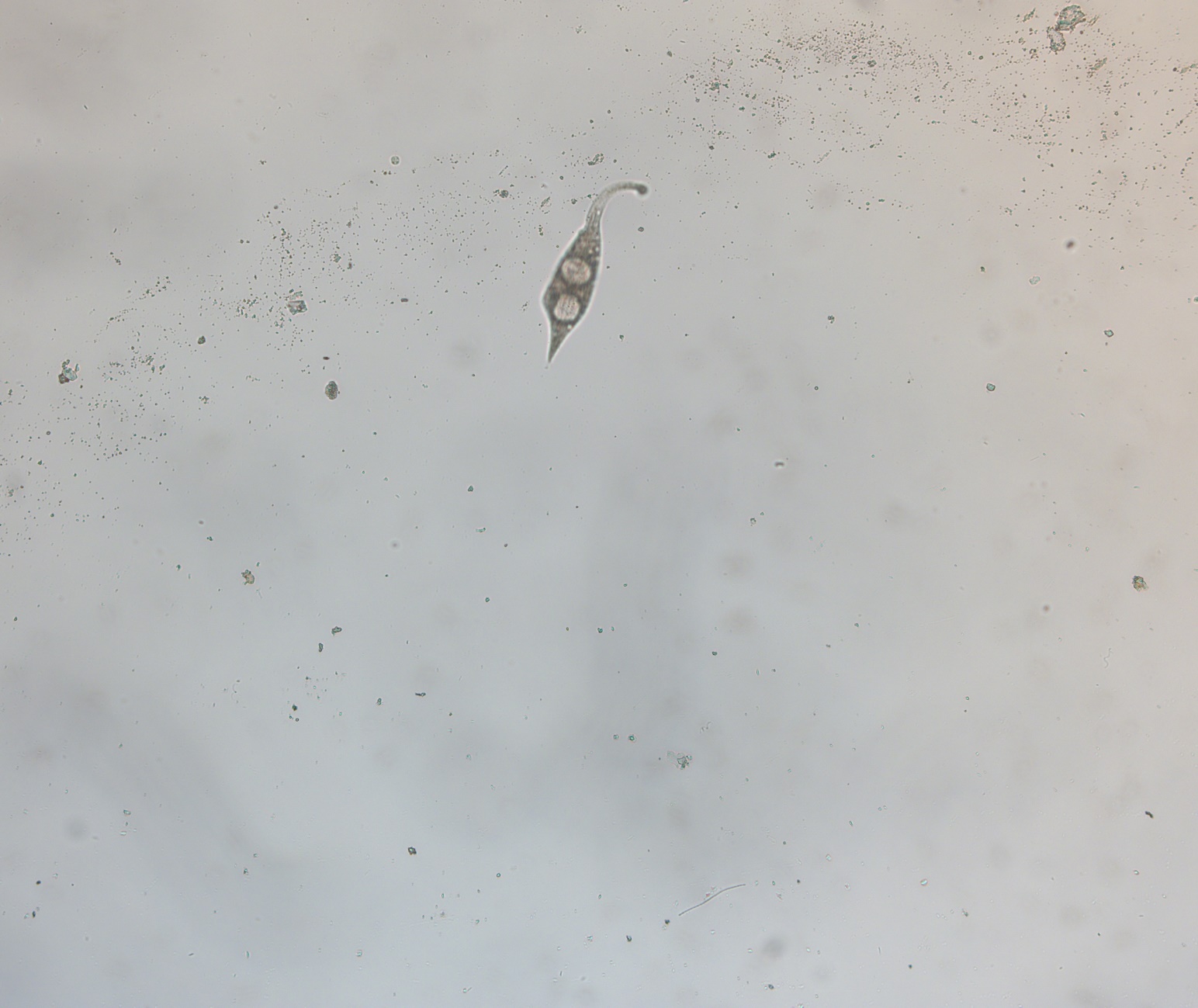


*Spathidium longicaudatum*


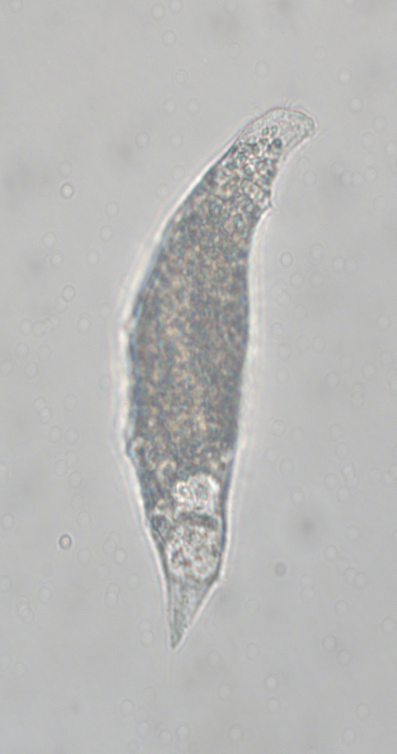


*Cyclidium versatile*


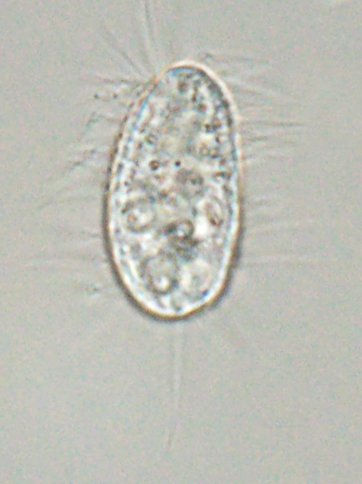


*Cyclidium simulans*


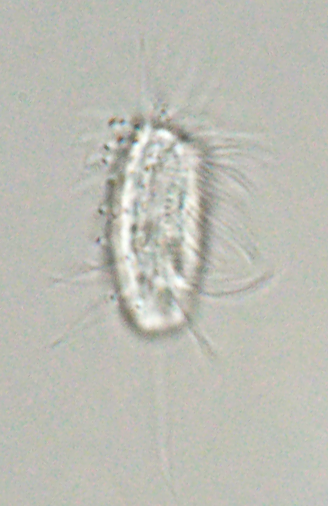


*Cyclidium centrale*


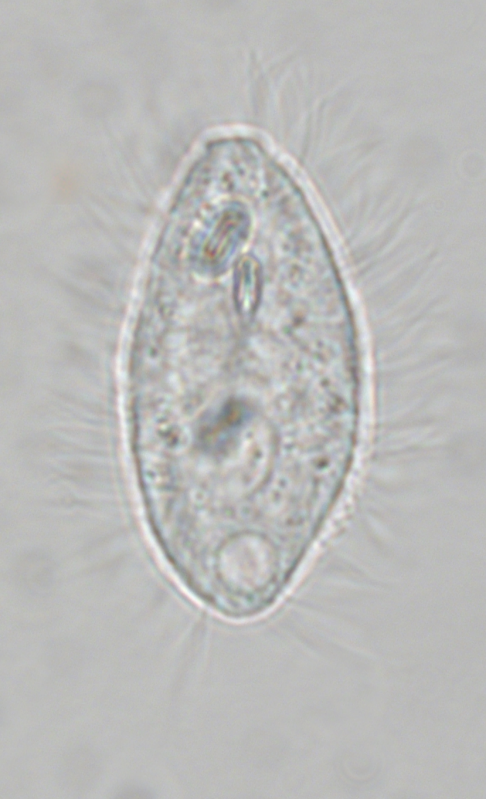


*Euplotes muscicola*


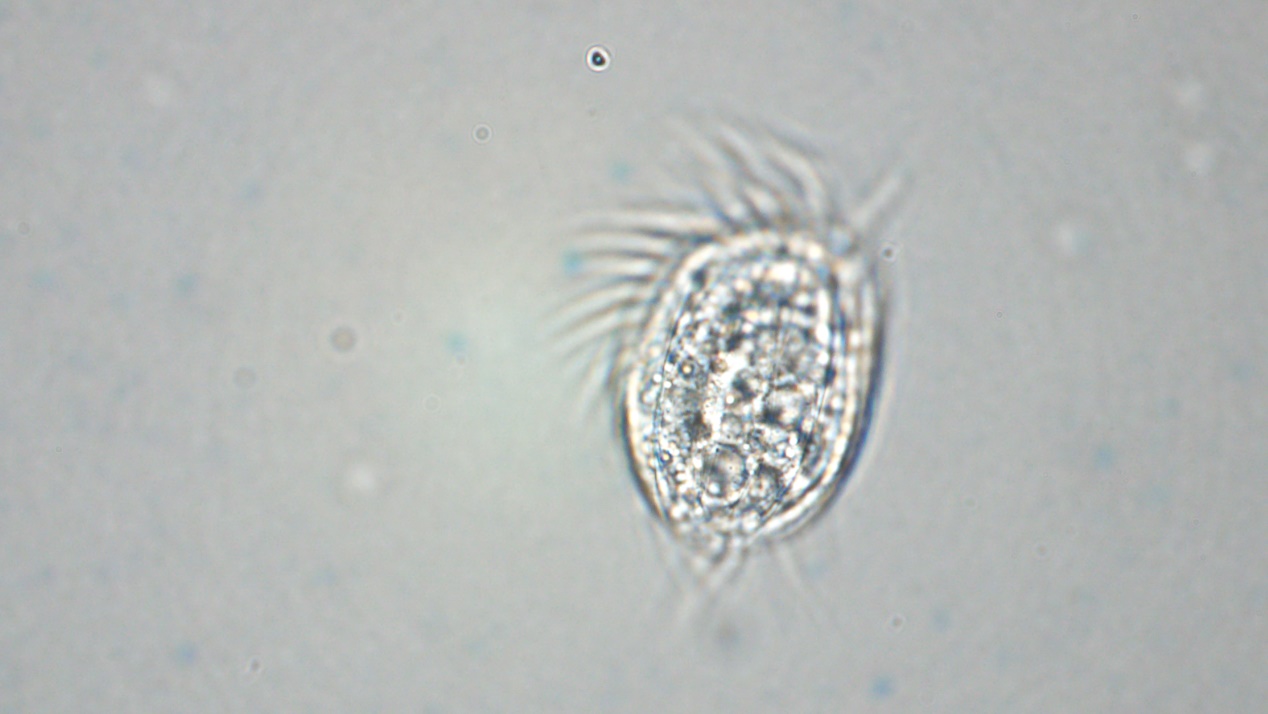


*Euplotes eurystomus*


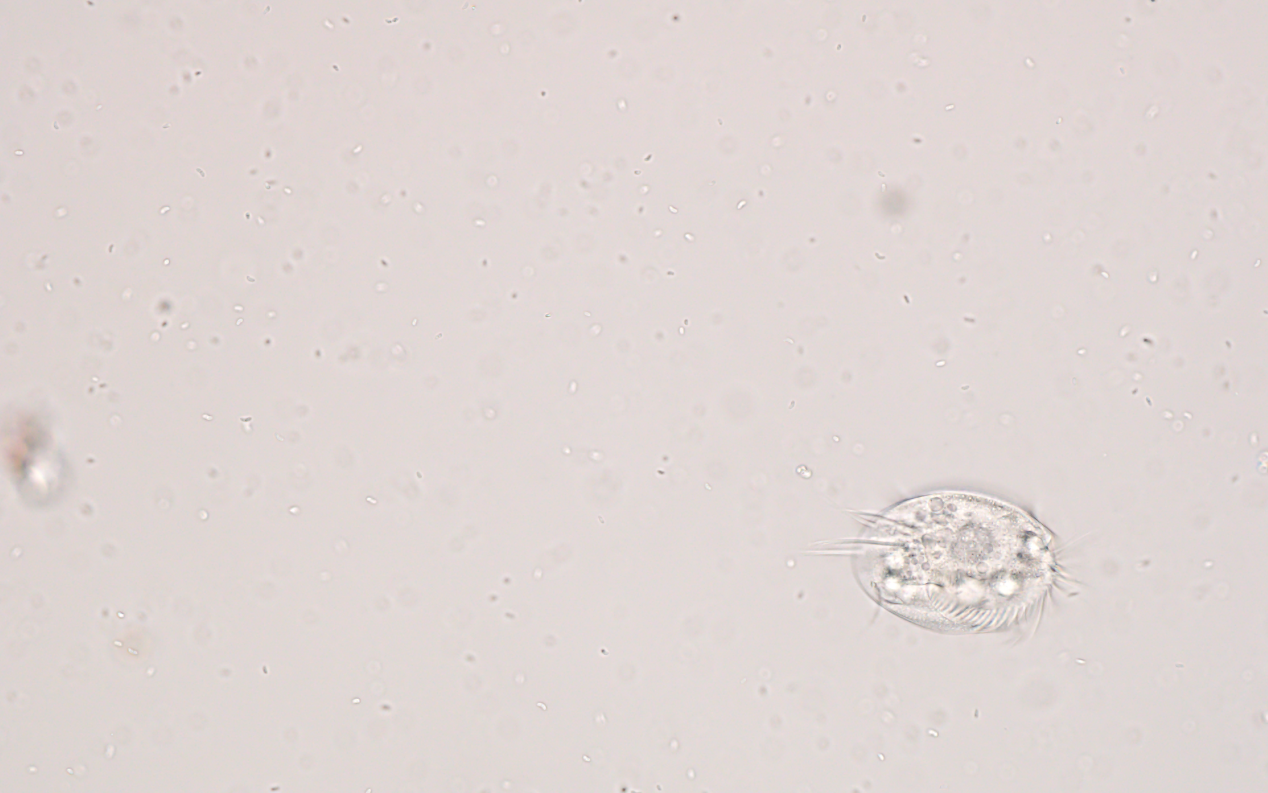


*Colpoda reniformis*
