# Supplementary material for: Soil water content drives the spatiotemporal the distribution and community assembly of soil ciliates in the Nianchu River Basin, Qinghai-Tibet Plateau, China
Source: PLoS One. 2024 Jul 10;19(7):e0299815. doi: 10.1371/journal.pone.0299815 (PMC11236148; doi:10.1371/journal.pone.0299815)
Supplement: Attachment — Submitted filename: Response to Reviewers.docx [file pone.0299815.s005.docx]

**Modification Description**

**Dear reviewer：**

Thank you very much for your comments on paper soil water content drives spatiotemporal the distribution and community assembly of soil ciliates in the Nianchu River Basin, Qinghai-Tibet Plateau, China (Manuscript ID：PONE-D-23-39236).Your valuable comments and suggestions are of great help to the revision and improvement of this article. The following is according to the teacher's opinion, we have made serious modifications. The blue font indicates the explanation, and the red font indicates the modification. Thank you!

**1.Since PLOS One is not a specialized ecology journal, definitions need to be provided for a broader audience. Examples- line 60 "community assembly",** **line 75 “driving factor and CONSTRUCTION MECHANISM’,** **line 79 “sustainable development of biodiversity”, line 135 “neutral community model” (what does this mean and what alterative models were tested?), line 196 “co-occurence network” (how do you distinguish between correlations and functionally cooperative relationships), deterministic versus random models (Discussion- L361). Alpha and beta diversity need to be defined.**

According to the reviewer’s comments, we have added explanations and changes to these contents.

line 60, changed “community assembly” to “community assembly (The process by which species settle and interact to establish and sustain local communities through successive repeated migrations from regional species banks)”

line 75, changed “driving factors and construction mechanism” to “driving factors (factors affecting soil ciliate community) and community assembly mechanism”

line 79, changed “sustainable development of biodiversity” to “sustainable development of biodiversity (biodiversity is the basis, goal and means of sustainable development)”

line 135, we explain the neutral model as follows: Developed by sloan et al., it is widely used in microbial community analysis. It establishes the hypothesis that "randomness, birth, death, and migration play a key role in shaping the structure of these communities" by quantifying neutral processes. By fitting the relative abundence-frequency relationships of different groups observed in microbial studies to beta distributions derived from neutral theory. The model has been shown to be able to successfully replicate the importance of species abundance distribution observed in large biomes.

line 196, we make the following explanations and distinctions:

correlations：we use the values of positive correlation ratio and negative correlation ratio to express them.

functionally cooperative relationships: the positive correlation in functional cooperation is solidarity cooperation and co-evolution. The negative correlation in functional cooperation is competition and predation. This part of the content cannot be reflected in the collinear network, but can be further explained by the positive and negative correlation between the network nodes.

L361, “changed deterministic processes” to “deterministic processes (interspecies interaction and environmental factor)”

Changed “random processes” to “random processes (birth, death, immigration, speciation and limited dispersal) ‘

Alpha and beta diversity need to be defined, changed “α and β diversity” to “α and β diversity (α diversity: the diversity of species in a habitat or community. β diversity: measures the rate of change of species composition from one community to another along a gradient at a regional scale)”

**2.** **Materials and Methods were sufficiently detailed, and the reference to the Lynn classification of ciliate species was sufficient. Since the classification of species in the different plots is central to the research, I agree with reviewer #2 that imaging data needs to be shown in supplemental data. An image of each species identified in the study should be provided for the different methods used to make your classification. The C/P quotient is defined sufficiently but I am not sure what you mean by r-selected and k-selected species). It is not clear how the value obtained in samplings serves as an indicator of the overall health of the soil.**

According to the reviewer’s comments, we added the display imaging data of the species to upload in the form of supplementary material.

Our interpretation of r-selected and k-selected species is:

R- selected: choices that favor increasing the intrinsic growth rate are called R- selected. R- selected are pioneers of new habitats, more adaptable to the environment, but survival depends on chance, so they are "opportunistic" in a sense, prone to "sudden outbreaks and violent bankruptcies."

K- selected: choices that favor increased competitiveness are called K- selected. K- selected are defenders of a stable environment. In a certain sense, they are conservatives. When there is a disaster in the living environment, it is difficult to recover quickly, and if there are competitors to restrain them, they may tend to become extinct.

In the paper A huge, undescribed soil 489 ciliate (Protozoa: Ciliophpra) diversity in natural forest stands of Central Europe, Foissner W et al. gave a detailed description of the value obtained by C/P as an indicator of the overall health status of soil.

When C⁄P≤1, the environment in the soil habitat is relatively good. When C⁄P＞1, the environment in the soil habitat is relatively bad.

**3.** **Starting with Table 3 (see also Table 4, Figure 3) data are provided for 4 groups designated CD, NT, SD and SJ. It is not clear from the main text, table or figure legends what these 4 designations are. I probably spent an hour trying to figure it out and never succeeded. The significance of the study cannot be assessed without a clear description of what is being analyzed. I don’t believe the findings for these groups were addressed in the Discussion.**

According to the reviewer’s comments, we made changes to the pictures in the manuscript.

Changed “CD” to “Grassland”, changed “NT” to “Farmland”, changed “SD” to “Wetland”, changed “SJ” to Sea buckthorn forest”

The revised manuscript picture is:

Fig 1

Fig 2

Fig 3

Fig 4

Fig 5

Fig 6

Fig 7

Fig 8

**4.** **Shannon and Margalef models: when I read the Materials and Methods, it seemed that both models are trying to assess similar phenomena. However, the later model shows only one statistically significant difference (Margalef model) in panel C. Please elaborate more on what the two models are assessing and what the data tell you based on only one comparison yielding significant differences (and with only one method).**

According to the reviewer’s comments, we make the following reply.

Margalef index reflects community species richness：The abundance of species in a community or environment, and an index of species richness in a biome (or sample). The larger the index, the higher the species richness in the ecosystem. However, this index has some limitations and needs to be combined with other diversity indexes. The index takes into account only the number and individual abundance of species, not the function and niche of species. Its application can help us understand the stability of ecosystems, environmental change and ecological restoration effects.

Shannon-Wiener index reflects community species diversity based on the number of species. The index is concerned not only with species richness, but also with species evenness, so it is a more comprehensive response to community structure.

Based on data from a comparison that produces a significant difference, the following conclusions can be drawn: Shannon-Weiner diversity index of soil ciliate community was significantly different in different time and space. These results indicate that the distribution of soil ciliate community is not uniform in different time and space, and the complexity of the community is different.

The Margalef index of soil ciliate community had significant differences among plots, but no significant differences in seasons. These results indicated that the species richness of soil ciliate community was different between plots, and the species richness of soil ciliate community was the same in season.

**5.** **The work focuses on species identification (# of species) as a function of soil type (landscape) over time (3 seasons). Why isn’t any data presented on the density of each species? Are there statistically significant seasonal fluctuations in the ABUNDANCE of different species (akin to a summer bloom)? Are there any physical characteristics of the different plot types that could be drivers of changes in community composition- temperature, water content?**

According to the reviewer’s comments, we uploaded the species density data to the database and the resulting link is as follows: DOI:10.6084/m9.figshare.25183643

There are no statistically significant seasonal fluctuations in the abundance of different species.

We conducted one-way ANOVA for different types of environmental factors and found that (Fig 6): Elevation, soil organic matter, soil temperature and soil water content were significantly different among different plots, suggesting that they might be the driving factors of community composition change. However, through mantel test and further analysis of these environmental factors, we found that soil water content was the main environmental factor driving soil ciliate community change.

**6.** **There are numerous comments in the discussion that are presented as factual findings, but that in my opinion are more sweeping generalizations that combine factual data with personal opinion. Line 267- “soil ciliate resources are abundant”- what are these resources and why is higher biodiversity a marker for resource abundance? Lines 267-271: need to define what you mean by harsh and better soil quality. Lines 314-320 in Discussion on multiple physical parameters of soils seems to have contradictory statements in it. If not, I am just confused. Not sure if you are talking about biodiversity differences between plot types or lumping all ciliates together. Community Assembly lines 352-368. You need to define the term and better relate your data to the subject. Are your conclusions rigorously drawn from the data or are you expressing an opinion that your data is consistent with. Again- neutral model is discussed. Are there other models that you should consider?**

According to the reviewer’s comments,

Line 267 We reviewed the description of the article and found that our description was inaccurate. Changed “Compared with other soil ciliate diversity studies^[9,13]^, the number of soil ciliate species in this area is relatively large. These results indicate that soil ciliate resources are abundant in the Qinghai-Tibet Plateau.” to “Compared with the number of soil ciliate species in other studies^[9,13]^, the number of species in this area is relatively large. It indicates that the soil ciliate species resources are abundant in the Qinghai-Tibet Plateau.”

Lines 267-271. We explain harsh and better soil quality. Changed “This is because soil ciliates of Spirotrichea tend to live in soils with better soil environmental quality. Soil ciliates of Colpodea tend to live in harsher soil environments” to “This is because soil ciliates of Spirotrichea tend to live in soils with better soil environmental (The change of soil environment is relatively stable). Soil ciliates of Colpodea tend to live in harsher soil environments (The change of soil environment is relatively drastic)”

Lines 314-320 We explain the content of this part. In the discussion of soil physical and chemical factors, we concentrated all soil ciliates and conducted an overall analysis of the environmental factors affecting soil ciliates. Because the screened soil water content, total nitrogen and organic matter are correlated with different plots.

lines 352-368 We are redefining the terms for the content of Community Assembly. Changed “community assembly” to “community assembly (The process by which species settle and interact to establish and sustain local communities through successive repeated migrations from regional species banks)”

Changed “deterministic processes” to “deterministic processes (interspecies interactions and environmental factors)”

Change “random processes” to “random processes (birth, death, immigration, speciation and limited dispersal)”

Our conclusions are drawn strictly from the data, and the views expressed are consistent with our data.

The neutral community model (NCM) quantifies the importance of stochastic processes.

R^2^ represents the overall fit of the neutral community model.R^2^ The higher the R2 value, the closer to the neutral model, it means that the community construction is more affected by random processes and less affected by deterministic processes.

N describes the metacommunity size, which in the text is the total abundance of all species in each sample.

m quantifies mobility at the community level, and the smaller the m value, the more restricted the dispersal of species throughout the community.

Therefore, the neutral model is sufficient and no other model is needed.

**7.Node calculations are not described in sufficient detail in the methods- how was inter-module connectivity (Zi) and inter-module connectivity (Pi) calculated?- also what is the difference between these?**

According to the editor's comments, we have added the calculation formulas for Zi and Pi to the manuscript. Zi is the z-scores of the connectivity degree within modules of node i, and Pi is the participation coefficient calculated according to the connectivity degree between modules of node i.

$Z_{i}\boldsymbol{=}\frac{K_{i}-\bar{K_{si}}}{{\sigma K}_{si}}$⑴

$P_{i}=1-\sum_{s=1}^{N_{m}} \left( \frac{K_{si}}{K_{i}} \right)^{2}$ ⑵

Where, Z_i_ is the connectivity degree of node i module, K_i_ indicates the connectivity of module i of node, $\bar{K_{si}}$ is the average value of K_i_ of all nodes in module s where node i resides, ${\sigma K}_{si}$is the standard deviation of connectivity within the module of all nodes in module s where node i resides, P_i_ is the participation coefficient of node i, Nm is the number of modular, s indicates the module s, and K_is_ is the connectivity of node i in each module

**8.** **Please ensure that your manuscript meets PLOS ONE's style requirements, including those for file naming. The PLOS ONE style templates can be found at**

**https://journals.plos.org/plosone/s/file?id=wjVg/PLOSOne_formatting_sample_main_body.pdf and**

**https://journals.plos.org/plosone/s/file?id=ba62/PLOSOne_formatting_sample_title_authors_affiliations.pdf**

According to the editor's comments, we have made changes to the format of the manuscript.

**9.** **Did you know that depositing data in a repository is associated with up to a 25% citation advantage (https://doi.org/10.1371/journal.pone.0230416)? If you’ve not already done so, consider depositing your raw data in a repository to ensure your work is read, appreciated and cited by the largest possible audience. You’ll also earn an Accessible Data icon on your published paper if you deposit your data in any participating repository (**[**https://plos.org/open-science/open-data/#accessible-data**](https://plos.org/open-science/open-data/#accessible-data)**).**

According to the editor's comments, we store the data in a repository, forming a link as: DOI: 10.6084/m9.figshare.25183643

**10.** **We suggest you thoroughly copyedit your manuscript for language usage, spelling, and grammar. If you do not know anyone who can help you do this, you may wish to consider employing a professional scientific editing service.**

According to the editor's comments, We invited Dr. Alan Warren from the Natural History Museum to make changes to the language of the manuscript. And add it to the author sequence as a collaborator. This was agreed upon by all the authors. Alan Warren is an expert in the systematics and biodiversity of ciliated protists (ciliates). We have sent a request email to the editorial office to add Dr. Alan Warren to the author sequence.

**11. We note that the grant information you provided in the ‘Funding Information’ and ‘Financial Disclosure’ sections do not match.** **When you resubmit, please ensure that you provide the correct grant numbers for the awards you received for your study in the ‘Funding Information’ section.**

According to the editor's comments, We have resubmitted the project number of the scholarship.

**Funding:** Comprehensive Scientific Investigation Project of Biodiversity Survey and Maintenance Mechanism Evaluation in the "One River and Four Rivers" Basin (Zangcai Science Education Index (2021) No.1 and Zangcai Education Index (2019) 01); Supported by "High-level Talents Cultivation Program" for Graduate Students of Tibet University, No.2020-GSP-S042. There was no additional external funding received for this study.

**12.** **Please provide an amended statement that declares all the funding or sources of support (whether external or internal to your organization) received during this study, as detailed online in our guide for authors at http://journals.plos.org/plosone/s/submit-now. Please also include the statement “There was no additional external funding received for this study.” in your updated Funding Statement.**

According to the editor's comments, we made changes to the funding statement. Attach the revised funding statement to the cover letter.

**13.** **PLOS requires an ORCID iD for the corresponding author in Editorial Manager on papers submitted after December 6th, 2016. Please ensure that you have an ORCID iD and that it is validated in Editorial Manager. To do this, go to ‘Update my Information’ (in the upper left-hand corner of the main menu), and click on the Fetch/Validate link next to the ORCID field. This will take you to the ORCID site and allow you to create a new iD or authenticate a pre-existing iD in Editorial Manager. Please see the following video for instructions on linking an ORCID iD to your Editorial Manager account:** [**https://www.youtube.com/watch?v=_xcclfuvtxQ**](https://www.youtube.com/watch?v=_xcclfuvtxQ)

According to the editor's comments, we have provided the ORCID iD in the Editorial Manager and updated the author information.

**14.** **We note that Figure 1 in your submission contain map images which may be copyrighted. All PLOS content is published under the Creative Commons Attribution License (CC BY 4.0), which means that the manuscript, images, and Supporting Information files will be freely available online, and any third party is permitted to access, download, copy, distribute, and use these materials in any way, even commercially, with proper attribution. For these reasons, we cannot publish previously copyrighted maps or satellite images created using proprietary data, such as Google software (Google Maps, Street View, and Earth). For more information, see our copyright guidelines:** [**http://journals.plos.org/plosone/s/licenses-and-copyright**](http://journals.plos.org/plosone/s/licenses-and-copyright)**.**

According to the editor's comments, We contact the original copyright owner and provide a content license form.

I request permission for the open-access journal PLOS ONE to publish Fig1 under the Creative Commons Attribution License (CCAL) CC BY 4.0 (http://creativecommons.org/licenses/by/4.0/). Please be aware that this license allows unrestricted use and distribution, even commercially, by third parties. Please reply and provide explicit written permission to publish Fig1 under a CC BY license and complete the attached form.
